# Signalling crosstalk at the leading edge controls tissue closure dynamics in the *Drosophila* embryo

**DOI:** 10.1371/journal.pgen.1006640

**Published:** 2017-02-23

**Authors:** Raphaël Rousset, Fabrice Carballès, Nadège Parassol, Sébastien Schaub, Delphine Cérézo, Stéphane Noselli

**Affiliations:** Université Côte d’Azur, CNRS, INSERM, iBV, France; New York University, UNITED STATES

## Abstract

Tissue morphogenesis relies on proper differentiation of morphogenetic domains, adopting specific cell behaviours. Yet, how signalling pathways interact to determine and coordinate these domains remains poorly understood. Dorsal closure (DC) of the *Drosophila* embryo represents a powerful model to study epithelial cell sheet sealing. In this process, JNK (JUN N-terminal Kinase) signalling controls leading edge (LE) differentiation generating local forces and cell shape changes essential for DC. The LE represents a key morphogenetic domain in which, in addition to JNK, a number of signalling pathways converges and interacts (anterior/posterior -AP- determination; segmentation genes, such as Wnt/Wingless; TGFβ/Decapentaplegic). To better characterize properties of the LE morphogenetic domain, we sought out new JNK target genes through a genomic approach: 25 were identified of which 8 are specifically expressed in the LE, similarly to *decapentaplegic* or *puckered*. Quantitative *in situ* gene profiling of this new set of LE genes reveals complex patterning of the LE along the AP axis, involving a three-way interplay between the JNK pathway, segmentation and HOX genes. Patterning of the LE into discrete domains appears essential for coordination of tissue sealing dynamics. Loss of anterior or posterior HOX gene function leads to strongly delayed and asymmetric DC, due to incorrect zipping in their respective functional domain. Therefore, in addition to significantly increasing the number of JNK target genes identified so far, our results reveal that the LE is a highly heterogeneous morphogenetic organizer, sculpted through crosstalk between JNK, segmental and AP signalling. This fine-tuning regulatory mechanism is essential to coordinate morphogenesis and dynamics of tissue sealing.

## Author summary

Dorsal closure of the *Drosophila* embryo is used as a paradigm to study epithelial sealing and is related to wound healing. This vital process relies on the dorsal migration of the two lateral ectodermal sheets and is necessary for the protective epidermis to completely envelop the embryo. The row of cells located at the front of migration, called the leading edge, is the organizing center of the process, where key signaling pathways turn on specific gene expression. Here we used a genomic approach to identify new genes whose expression is restricted to the leading edge. A quantitative analysis revealed differential expression along the anterior-posterior axis of the leading edge, which was considered for a long time as homogeneous or amorphous. We demonstrate that anterior-posterior cues provide an orthogonal coordinate system specifying cell identity along the whole leading edge, making it a highly patterned morphogenetic center. We further show that these anterior-posterior cues are functionally important, controlling the dynamics of dorsal closure and participating to the robustness of the process. Our work sheds new light on the role of anterior-posterior cues in epithelial tissue sealing related to wound-healing.

## Introduction

Epithelial morphogenesis is orchestrated at the cellular level through local shape changes and tension-based dynamics. In this process, cell-cell signalling plays an essential role in coordinating gene expression programs with tissue behaviour. One of the best-studied morphogenetic movements is embryonic dorsal closure (DC) in *Drosophila*. Following germ band retraction, the embryo is only partly enveloped by the epidermis, leaving a large dorsal area covered by the transient squamous amnioserosa. DC uses a combination of signalling pathways to generate mechanical forces, cell shape changes and cell adhesion rearrangements causing the two lateral epidermal sheets to spread dorsalwards and fuse at the dorsal midline through a zipping mechanism.

At the onset of DC, the dorsal-most epidermal cells, a.k.a leading edge cells or LE cells, polarise under the influence of the canonical Wingless (Wg) pathway and elongate along the dorso-ventral axis [1–3]. LE polarization leads to a particular organization of the dorsal cell membrane (that in contact with the amnioserosa), which in particular loses adherens junction markers (such as E-Cadherin, ECad) and septate junction markers (such as Discs-large, Dlg) [2, 4, 5]. This redistribution results in the formation of actin-nucleating centers (ANC) from either side of the dorsal membrane that will transmit, along with the adherens junctions, mechanical forces [2, 5, 6]. LE cells start synthesising an ANC-linked acto-myosin cable that generates a contractile force allowing their alignment during dorsal migration [7, 8]. The acto-myosin cable also participates in a ratchet-like process induced by the asynchronous pulsation of the amnioserosa cells [9]. By progressively contracting and disappearing by cell engulfment and apoptosis, the amnioserosa supplies an additional positive force, whereas the stretching lateral ectoderm opposes a resistance force to tissue progression [8, 10]. The two contralateral ectodermal sheets then adhere to each other starting from the two canthi of the hole in a process called zipping, providing a fourth force to DC [6, 8, 11]. Non-muscle myosin II is involved in the generation of the four forces thanks to its motor and contractile activities [12].

The LE is the organizing centre of DC and is specified by JNK signalling. Mutations in components of the JNK pathway such as *DJNKK/hemipterous (hep*) or *DJNK/basket* (*bsk*) strongly affect DC, leaving embryos with dorsal holes in their cuticle [1, 13, 14]. JNK signalling is specifically activated in the LE cells, controlling the expression of the target genes *puckered* (*puc*, as revealed by the *lacZ*-containing enhancer-trap *puc-Z*), *decapentaplegic* (*dpp*) and *scarface* (*scaf*) [1, 15–17]. Recently, a feed-forward loop between the JNK and Dpp activities was shown to regulate the expression in the LE of three proteins, the myosin VI homologue Jaguar, the microtubule-binding protein Jupiter and the integrin-linked Zasp52 [18]. Other JNK target genes, whose expression is not limited to the LE during DC, have also been described, such as the profilin-coding gene *chickadee*, the transcription factor *cabut*, the integrin-coding genes *scab* and *myospheroid*, and the trafficking gene *Rab30* [19–22].

A specific feature of DC is that it occurs in a field of cells that is not uniform along the anterior-posterior (AP) axis, encompassing the thoracic and abdominal regions. In addition, the ectodermal cells (LE and lateral ectoderm) are divided in repeated, segmental units (T1-T3 and A1-A8 segments). Whereas the HOX genes define the identity of the segments along the AP axis [23], the segment polarity genes are responsible for the elaboration of cellular patterning within each segment of the *Drosophila* embryo leading to the formation of anterior and posterior compartments [24]. Therefore, it is important to characterize the interaction between these orthogonal signalling events (JNK vs. AP/segmentation signalling) and determine how this interaction controls tissue morphogenesis.

Previous studies have shown that the Wg pathway collaborates with JNK to induce *dpp* expression in the LE [3, 25]. It was also demonstrated that the dynamics of closure presents robust asymmetric properties along the AP axis [10, 26]; for instance, the anterior speed of closure is faster than the posterior one, which could be due to localized apoptotic forces present in the anterior amnioserosa [10]. Yet, whether and how segmentation and AP cues impact on DC is currently unknown. In this study, we characterized the genomic response of the JNK pathway during DC, revealing a whole set of new target genes, several of them being specifically transcribed in the LE. Quantification of these new intra-LE expression profiles uncovers a complex organization of the LE that depends on crosstalk between JNK, HOX and segmentation pathways. In this network, HOX genes can have positive or negative activities, regulating segmental features during closure. For instance, loss of the posterior HOX gene *abdominal-A* (*abd-A*) or *Abdominal-B* (*Abd-B*) leads to closure delays of the most posterior segments, indicating that they control the timing of closure. Thus, crosstalk between the AP and JNK systems shapes the LE organizing centre for proper tissue sealing dynamics.

## Results

### JNK transcriptional response during DC

**Fig 1:**
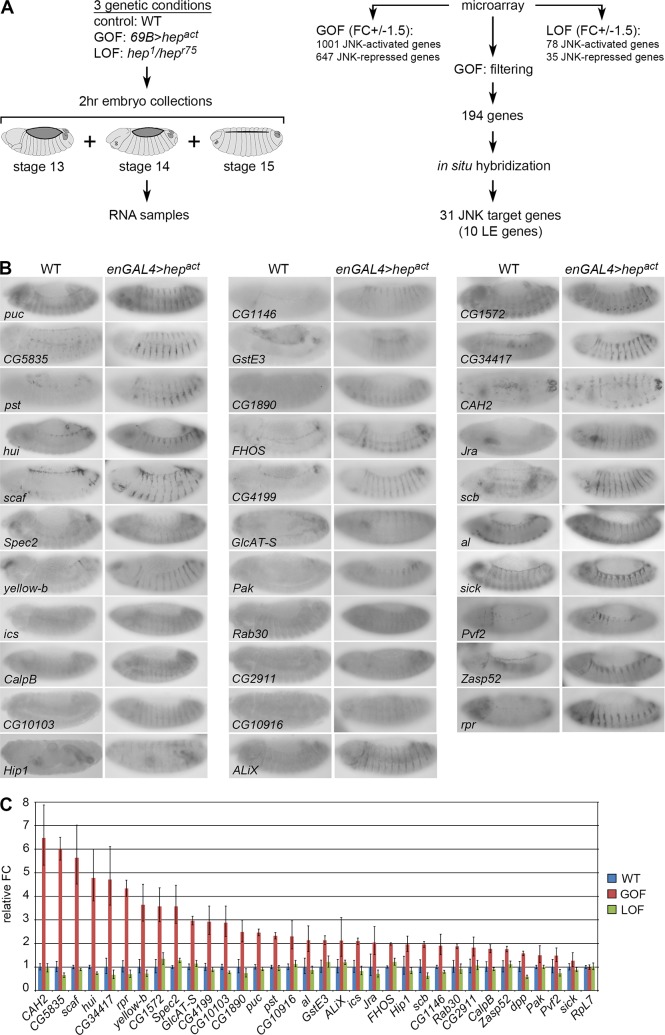
Identification of new JNK target genes during DC. **A)** Phases of the genomic screen (see text for details). **B)** *In situ* hybridizations of WT (1^st^, 3^rd^ and 5^th^ columns) and *en-GAL4 > hep*^*act*^ (2^nd^, 4^th^ and 6^th^ columns) embryos undergoing DC. Target gene up-regulation is induced in stripes upon ectopic activation of the JNK pathway using an *en-Gal4* driver. **C)** Analysis of JNK target gene expression using quantitative PCR in WT (*w*^*1118*^, blue), GOF (*69B-GAL4 > hep*^*act*^, red) and LOF (*hep*^*1*^*/hep*^*r75*^, green) conditions. The same RNA samples as those used for microarray analysis were used. *puc* and *dpp* were used as positive controls. All genes, except the negative control *RpL7* (1.0), show an up-regulation of their expression in GOF embryos with a relative fold change between 1.3 (*sick*) and 6.5 (*CAH2*). Y-axis represents the relative fold change (FC) +/- s.d..

To better characterize the LE and its role during DC, we used microarrays to identify genes expressed under the control of the JNK pathway in the *Drosophila* embryo (Figs 1A and S1A). Three different conditions for JNK activity were tested: wild-type (WT; used as reference), loss-of-function (LOF) and gain-of-function (GOF). GOF embryos were obtained by ectopically expressing the active form of the DJNKK/Hep (Hep^act^) protein in the ectoderm using the *69B-GAL4* driver, while LOF embryos were generated using a *hep*^*r75*^ / *hep*^*1*^ hetero-allelic combination [1]. The extent of JNK activity and sample homogeneity in the three groups of embryos were assessed through *in situ* hybridization using a *dpp* probe (S1A Fig).

Total RNAs were prepared from carefully-staged embryos and analysed by microarray. The statistical comparison of WT and GOF conditions identified 1648 independent genes (corresponding to 1679 probes) which are regulated by a factor equal to or higher than 1.5, of which 1001 (1023 probes) are activated by JNK and 647 (656 probes) are inhibited (S1B Fig). For the WT-LOF comparison, 113 distinct genes (117 probes) are uncovered with a fold change limit of 1.5, with 35 (37 probes) activated in the *hep* mutant (i.e. inhibited by JNK) and 78 (80 probes) inhibited (S1B Fig).

These results revealed a smaller number of genes controlled in LOF compared to GOF. This difference is not surprising when comparing the extent of JNK activity (evaluated through the expression of *dpp*) in GOF/LOF vs. wild-type embryos. Indeed, JNK activity is strongly increased in GOF embryos, while only LE cells (representing approximately a mere 200 cells of the whole embryo) lose *dpp* mRNA in LOF embryos (S1C Fig). This difference in amplitude between GOF and LOF likely explains the fact that only a few genes are found in common between the two lists (S1 Table). For this reason, we focused on the WT-GOF comparison, and in particular on the genes that are activated by the JNK pathway.

We initially performed a functional classification of 1648 GOF genes based on Gene Ontology (GO) terms, using the David bioinformatics tool which has the advantage of making family groups from GO terms and ranking them according to enrichment scores [27]. Functional classification of the 1001 up-regulated genes (S2 Table) and 647 down-regulated genes (S3 Table) revealed that during DC, JNK signalling controls a wide range of biological functions including cell cycle, cytoskeleton, proteolysis, mRNA transport, and regulation of transcription.

### Identification of 31 JNK target genes

**Fig 2:**
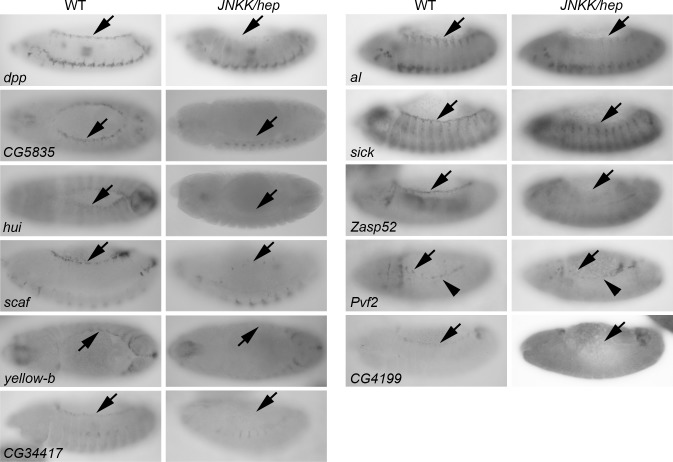
New genes showing JNK-dependent expression in the LE. *In situ* hybridizations showing expression in the LE of *dpp* (control) and of the 10 LE genes (1^st^ and 3^rd^ columns; arrows). LE-specific expression is lost in *hep* mutant embryos (2^nd^ and 4^th^ columns; arrows), indicating that the JNK pathway drives LE expression of these genes. Of note, expression is not uniform along the whole LE.

To further identify *bona fide* JNK target genes, we performed *in situ* hybridization on *en-GAL4/UAS-hep*^*act*^ embryos, in which the JNK pathway is activated in a striped pattern, as seen with the *puc* control (Fig 1B). Because of the great number of identified genes, we first made a selection of genes based on their potential localization and function (membrane, secreted, motors, etc…) and on available expression data from public databases (such as FlyExpress). In addition, comparison of our embryonic data set with *Drosophila* Schneider-2 cells induced by the lipopolysaccharide JNK activator, allowed the extraction of a list of genes having a common transcriptional response to JNK activity [28, 29]. In total, we selected and tested 194 genes by *in situ* hybridization and identified 31 *bona fide* JNK target genes (Fig 1B; S4 Table). The response of these 31 genes to JNK signalling was further confirmed by quantitative RT-PCR (Fig 1C). Of the 31 genes identified, some have already been described as transcriptional targets of the JNK pathway (*jra/jun*, *scab*, *Zasp52*, *scaf*, *Rab30* and *reaper*) [17, 18, 21, 22, 30, 31], while others have been linked to the JNK pathway and/or to DC without being described as transcriptional targets of JNK (*Pak*, *icarus* and *ALiX*) [32–34], thus validating our approach and genomic data. Interestingly, 10 out of 31 genes show specific expression in the LE, which is dependent on JNK activity as their expression is lost in the *hep* mutant, similarly to *puc* or *dpp* (Fig 2). Therefore, transcriptional profiling of embryos undergoing DC allowed the identification of several new JNK target genes (representing 25 genes plus the previously known ones: *jra/jun*, *scab*, *Zasp52*, *reaper*, *scaf* and *Rab30*; i.e. 31 genes in total), including 10 (8 new in addition to *scaf* and *Zasp52*) with a specific expression in the LE, like the well-known LE JNK target genes *puc* and *dpp* (hereafter referred to as “LE genes”).

### Spatio-temporal heterogeneity of gene expression at the LE

**Fig 3:**
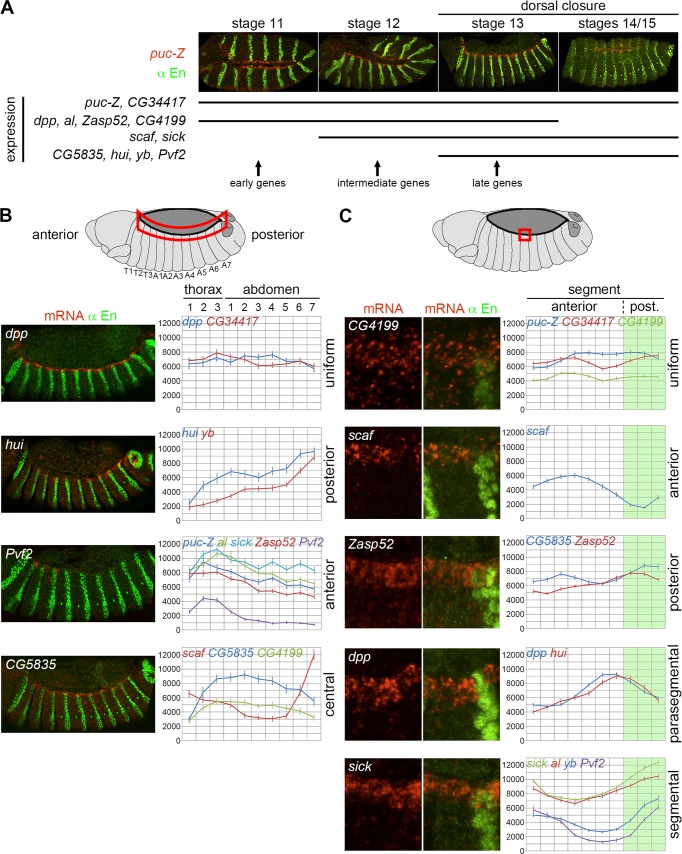
Spatio-temporal regulation of JNK target gene expression at the LE. **A)** Temporal expression (black lines) of the twelve LE genes from stage 11 (full extension of the germ band) to stages 14/15 (end of DC), including stage 12 (germ band retraction) and stage 13 (onset of DC). Expression was monitored in WT embryos using FISH-IF, as shown with *puc-Z/+* embryos (upper panels; *lacZ* mRNA in red and anti-En staining in green). **B)** Quantitative analysis of the expression of the twelve LE genes along the AP axis of the fly embryo (right panels), with one representative gene (FISH-IF, left panels) for each of the four categories identified: uniform (*dpp*, *CG34417*), posterior (*hui*, *yb*), anterior (*puc-Z*, *al*, *sick*, *Zasp52*, *Pvf2*) and central (*scaf*, *CG5835*, *CG4199*). LE mRNA quantification from FISH-IF experiments is based on the average fluorescent intensity in each segment from T1 to A7, as indicated by the red area on the depicted embryo (top panel). Expression is indicated as fluorescent intensity (a.u. +/- s.e.m.). n = 20 embryos, except for *hui* (n = 15) and *yb* (n = 16). **C)** Segmental expression of the LE genes. Each FISH-IF panel (left panels) displays one representative example of the five groups: uniform (*puc-Z*, *CG34417*, *CG4199*), anterior (*scaf*), posterior (*CG5835*, *Zasp52*), parasegmental (*dpp*, *hui*) and segmental (*sick*, *al*, *yb*, *Pvf2*). On average, the segments are formed by ten cells of which seven are anterior (white) and three posterior (green). mRNA signal quantification (right panels) for a given gene is based on the average fluorescent intensity (a.u. +/- s.e.m.) in each of the ten cells of all the segments taken together.

Surprisingly, we noticed that the expression of most of the LE JNK target genes was not spatially and temporally homogeneous along the LE. First, the onset and arrest of gene expression show temporal regulation during DC. Genes can start to be expressed from either stage 11 (corresponding to full germ-band extension; *puc-Z*, *CG34417*, *dpp*, *al*, *Zasp52*), stage 12 (during germ-band retraction; *scaf*, *sick*) or at the onset of DC at stage 13(*CG5835*, *hui*, *yb*) (Fig 3A). Termination of expression also varies from gene to gene. The expression of *dpp*, *al* and *Zasp52* decreases very rapidly after the start of DC and is no longer visible at stage 14 while DC is still in progress. In contrast, other genes lose their LE expression after the complete fusion of the lateral sheets of the epidermis. To our knowledge, this is the first time that early, intermediate and late JNK-target gene expression is described in a developmental process (Fig 3A), whereas it is known that the JNK pathway has distinct phases of strength and duration [35–38]. Second, our initial *in situ* hybridization experiments suggested heterogeneous expression along the LE (Fig 2). In order to precisely define the expression pattern of each LE gene, we developed CurvedPeriodicity, a program to quantify the mRNA levels specifically in the LE from fluorescent *in situ* hybridizations (FISH). The FISH experiments were coupled to immuno-fluorescence (FISH-IF) revealing the En protein to delineate the LE borders and position the segment boundaries. We extracted from CurvePeriodicity the fluorescence intensity per segment, showing the existence of 4 intra-LE profiles with either uniform expression along the entire length of the LE (*dpp* and *CG34417)*, or expression domains biased towards the anterior (*puc-Z*, *al*, *sick*, *Zasp52* and *Pvf2*), posterior (*hui* and y*b*) or central (*scaf*, *CG5835*, *CG4199*) portions of the LE (Fig 3B). *Pvf2* is a remarkable example of the ‘anterior’ group, being only expressed in the three thoracic segments (posterior expression in Fig 2 corresponds to cardiac cells). In the central domain, expression can be either lowered (*scaf*) or increased (*CG5835*, *CG4199*) compared to more terminal parts.

In addition to this global LE heterogeneity along the AP axis, we also found striking differences in the segmental expression of JNK target genes (Fig 3C). Quantitative analysis revealed five different categories of segmental profiles: i) a linear profile, with no significant change along the segment (*puc*, *CG34417*, *CG4199*), ii) anterior and iii) posterior profiles, showing stronger expression in the anterior (*scaf*) or posterior (*CG5835*, *Zasp52*) compartment, respectively, iv) a parasegmental boundary profile in which highest expression is observed between the anterior and posterior compartments of the same segment (*dpp*, *hui*) and v) a segmental boundary profile in which highest expression is found at the segment boundary (*sick*, *al*, *yb*, *Pvf2*). Altogether, these results identify a highly complex, spatio-temporal regulation of the JNK transcriptional response during DC both along the anterior-posterior axis and within segments, raising the question of the mechanisms setting these different transcriptional programs at the LE.

### The segmentation gene *en* is a repressor of LE gene expression

**Fig 4:**
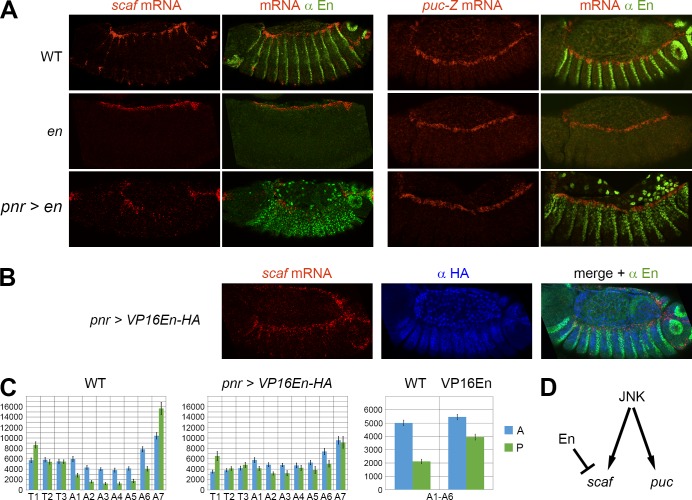
En is a direct repressor of *scaf* expression. **A)** FISH-IF showing *scaf* (left panels) or *puc-Z* (right panels) expression in WT embryos (top panels), *en* mutants (middle panels) and embryos overexpressing *en* with *pnr-GAL4* (lower panels). The segmented expression of *scaf* is lost in the *en* mutant, and *scaf* is down-regulated in *en*-overexpressing embryos. In contrast, no change is observed with the non-segmented expression of *puc-Z*. The *pnr-GAL4* driver induces homogenous ectopic expression of *en* in the dorsal part of the embryo (dorsal ectoderm and amnioserosa), as indicated by the anti-En staining (green). **B)** and **C)** The repression domain of En is required for the inhibition of *scaf* expression. **B)** FISH-IF showing that the overexpression with *pnr-GAL4* of the VP16En mutant form, for which the repressor domain has been replaced by VP16, does not inhibit *scaf* expression. Anti-HA immunostaining (blue) was used to select embryos overexpressing VP16En, which is HA-tagged in its N-terminal part. **C)** Quantification of *scaf* expression in WT or VP16En-overexpressing embryos. The two bar diagrams on the left show the expression intensity (a.u. +/- s.e.m.; n = 20 for WT and n = 15 for *pnr > VP16En*) for each compartment (anterior in blue and posterior in green) in each segment. The bar diagram on the far right represents the average expression in the central A1 to A6 segments of WT or VP16En-overexpressing embryos, which shows an increase of *scaf* mRNA in the posterior compartments with VP16En. **D)** Summary of the regulatory network occurring in the LE.

To address this question, we used the JNK target gene *scaf* as readout because it exhibits a strong expression in the LE that undergoes both AP and segmental regulations (Figs 3B, 3C and 4A). Quantification in the A and P compartments from each segment revealed that *scaf* is down-regulated in the P compartments of the central abdominal segments, from A1 to A6 (Fig 4C). The posterior factor En has been shown to have both activating and repressive transcriptional activities [39], making it a potential *scaf* regulator in this process. In *en* null mutant embryos, *scaf* expression is no longer segmented (Figs 4A and S4A). Moreover, the overexpression of *en* with the *pannier* (*pnr*)-GAL4 driver in the whole dorsal ectoderm leads to down-regulation of *scaf* expression, but strikingly, this inhibition only occurs in the abdominal segments. Of note, *en* had no influence on the non-segmented expression of the *puc-Z* JNK reporter gene (Fig 4A). Altogether, these results indicate that *en* is a LE negative regulator, repressing the posterior expression of a subset of JNK target genes, including *scaf*. To assess if En acts directly or through a relay mechanism, we expressed a mutant form of En in which the repressor domain has been replaced by the VP16 transactivation domain (VP16En) [39]. In contrast to the wild-type form, the overexpression of VP16En has no inhibitory effect on *scaf* expression, supporting a direct repressive activity of En (Fig 4B and 4C). This result also indicates that the effect of *en* occurs independently of the auto-regulatory loop involving *wg* and *hh*. Additionally, we observed that in VP16En-overexpressing embryos, the *scaf* expression level in the posterior compartments of central segments is higher than in WT embryos, indicating that VP16En can compete with endogenous En (Fig 4C). Altogether these results indicate a direct role of the segment polarity gene *en* in patterning the LE during DC, shown by the control of the JNK target gene *scaf* (Fig 4D).

### The HOX gene *abd-A* collaborates with *en* to repress *scaf* expression

**Fig 5:**
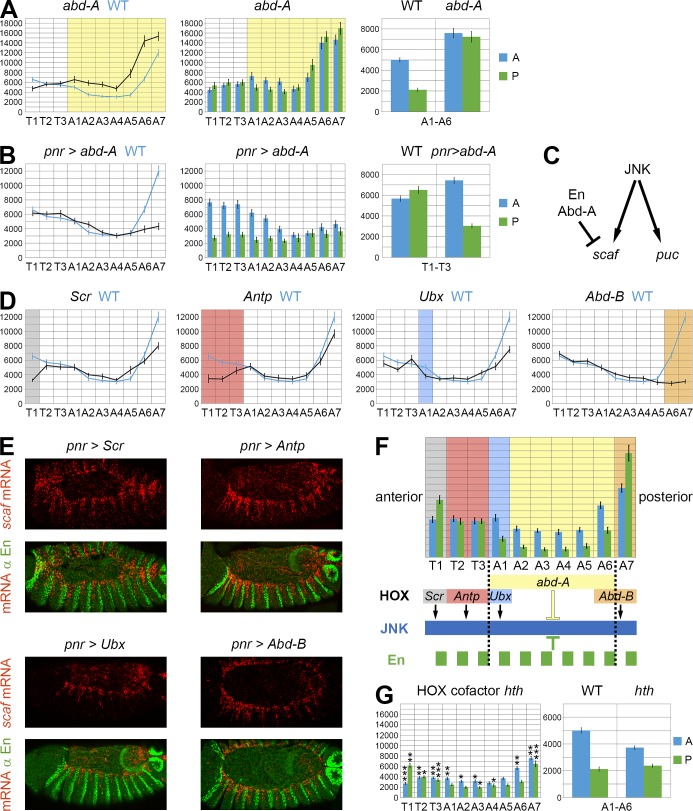
HOX regulation of *scaf* expression. **A)** Quantification of *scaf* expression in the *abd-A* mutant (n = 15). First bar diagram: expression along the AP axis of the *abd-A* mutant (black) is higher than in WT embryos (blue), but only in the abdominal segments where *abd-A* is active (yellow). Second bar diagram: compartmental expression of *scaf* (anterior in blue and posterior in green) showing that posterior compartment expression is highly increased compared to WT embryos (shown in Fig 4C, first panel). Third bar diagram: average expression in the central A1 to A6 segments of WT (see Fig 4C) and *abd-A* embryos, which shows the same level of expression in the A and P compartments in the *abd-A* mutant. **B)** Quantification of *scaf* expression in embryos overexpressing *abd-A* (*pnr > abd-A*; n = 20). First bar diagram: compared to WT embryos (blue curve), overexpression of *abd-A* (black curve) leads to *scaf* mRNA down-regulation in the posterior region. Middle bar diagram: *abd-A* overexpression also induces *scaf* mRNA diminution in the posterior compartments (i.e. *en*-expressing cells) of thoracic segments, where Abd-A is normally absent, indicating that Abd-A requires En for its repressive function. Right bar diagram: average expression in the thoracic segments T1-T3 of WT embryos (from Fig 4C) and *abd-A*-overexpressing embryos. **C)** Summary of the regulatory network occurring in the LE. **D)** *scaf* expression (black curves) in *Scr* (first panel), *Antp* (second panel), *Ubx* (third panel) and *Abd-B* (fourth panel) mutants (n = 15 for each) compared to WT embryos (blue curve). Each main expression domain of the corresponding HOX gene is indicated by a color: grey for *Scr*, pink for *Antp*, blue for *Ubx* and orange for *Abd-B*. Reduction of *scaf* expression is restricted to the main domain of HOX gene activity. **E)** FISH-IF showing the ectopic expression of *scaf* in embryos overexpressing HOX genes in the dorsal ectoderm with *pnr-GAL4*: *Scr* (top left panels), *Antp* (top right panels), *Ubx* (lower left panels) and *Abd-B* (lower right panels). **F)** Summary of the regulatory network controlling LE expression. *scaf* expression is regulated by the combinatorial activity of the JNK pathway (specific of the LE), *en* (posterior compartments) and the HOX genes (AP cues). Whereas *Scr*, *Antp*, *Ubx* and *Abd-B* act as positive regulators, *abd-A* and *en* collaborate to antagonize *scaf* expression. **G)** Quantification of *scaf* expression in the *hth*^*P2*^ mutant (n = 16), showing that the cofactors Hth and Exd are necessary for the regulation by the HOX genes (left panel). The one-way test (test for Equal Means in a One-Way Layout) with Monte Carlo resampling was done using R to compare with WT embryos (shown in Fig 4C): *: p < 0.05; **: p < 0.01; ***: p < 0.001. In the right panel, only the segments from A1 to A6 were quantified, averaged and compared to WT embryos (see Fig 4C). In all quantification panels, Y axis corresponds to fluorescent intensity (a.u. +/- s.e.m.).

Despite being expressed in all segments, the repressor activity of En is restricted to central abdominal segments, indicating the existence of additional regulatory mechanisms to circumscribe En action and refine LE patterning. We thus tested the role of the HOX genes, which control segment identity along the AP axis [23]. The HOX gene *abd-A* is expressed and acts in the abdominal segments. In *abd-A* null mutant embryos, the expression of *scaf* is increased in segments A1-A7 (i.e. in the *abd-A* domain; Figs 5A and S4B), indicating that *abd-A* negatively regulates *scaf*. Interestingly, quantification of *scaf* expression at the segmental compartment level shows that it is no longer repressed in the P compartments of central segments (A1-A6). This *abd-A* mutant phenotype is therefore similar to the *en* mutant phenotype, suggesting that both genes work together to control *scaf* in the LE. When overexpressed, *abd-A* leads to decreased *scaf* expression in the posterior segments (Figs 5B and S4C). In the thoracic segments where *abd-A* is normally not expressed, we observed *scaf* down-regulation in the P compartments where *en* is also present, indicating that the inhibition of *scaf* expression in the P compartment requires both En and Abd-A. This phenotype explains why the overexpression of En has a restricted effect in the abdominal segments where *abd-A* is expressed, with no effect in the thoracic segments where *abd-A* is absent (Fig 4A). Therefore, En and Abd-A collaborate to down-regulate *scaf* in the central segments, where their pattern of expression overlaps. In conclusion, En and Abd-A are two repressors regulating LE gene expression (Fig 5C).

### The HOX genes *Scr*, *Antp*, *Ubx* and *Abd-B* are activators of *scaf* expression

We extended our study to the other HOX genes that are expressed in the dorsal ectoderm during DC (Fig 5F) by testing the role of *Sex combs reduced* (*Scr*; expressed in the T1 segment), *Antennapedia* (*Antp*; expressed in the T1-T3), *Ultrabithorax* (*Ubx*; strongest expression in A1, declining until A7) and *Abd-B* (strongest expression in A8, declining towards A6) [23]. Null mutants for either Hox gene showed reduced *scaf* mRNA levels in their expression domain, suggesting they all have a positive role on *scaf* transcription (Figs 5D and S4D). Consistently, upon overexpression, these HOX genes were all able to ectopically up-regulate *scaf* expression (Fig 5E). Therefore, in contrast to *abd-A* which acts as a repressor, *Scr*, *Antp*, *Ubx* and *Abd-B* activate the expression of *scaf* in their respective functional domain.

All these results reveal that key transcription factors controlling AP axis and segmentation work together generating a complex gene regulatory network to control gene expression along the LE. This LE network, which involves both activating (JNK, *Scr*, *Antp*, *Ubx* and *Abd-B*) and repressive (*abd-A* and *en*) activities, leads to a remarkable transcription profile of *scaf* in the LE, with high expression at the poles of the embryo and low expression in the P compartments of A1-A6 segments (Fig 5F).

### HOX cofactor requirement for LE gene expression

HOX proteins cooperate with cofactors to activate transcription [40]. Homothorax (Hth) and Extradenticle (Exd) are known to interact with each other and with HOX proteins to form a transcriptional complex binding specific cis-regulatory sequences [41]. Hth contains a homeodomain (HD) in its C-terminal part that is responsible for DNA binding. In addition, Hth promotes Exd nuclear translocation and its binding to DNA [40, 42–44]. In order to inquire the role of HOX cofactors on *scaf* expression, we thus analysed embryos mutants for *hth*^*P2*^, a strong hypomorphic allele resulting in an absence of Hth protein expression [45]. As a result, Exd cannot be translocated into the nucleus. In absence of cofactor function, *scaf* expression decreases (Fig 5G). This is especially the case in the more extreme segments, whereas the effect is only moderate in the central segments. Indeed, we still observe in the region A1-A6 of the *hth*^*P2*^ mutant the segmented pattern of *scaf* expression, with the same level of transcripts in the posterior compartments than wild-type embryos (Fig 5G, right panel and S4E Fig). These results indicate that the HOX cofactors Hth and Exd are required for the positive regulation of *scaf* expression in the LE by Scr, Antp, Ubx and Abd-B, but do not participate in the negative action of En and Abd-A. Therefore, in addition to the core LE network involving the transcription factors AP1, HOX and En, the HOX cofactors have a distinctive contribution along the AP axis according to the identity of the segments, further refining LE patterning.

### HOX genes are important regulators of JNK-dependent LE patterning

**Fig 6:**
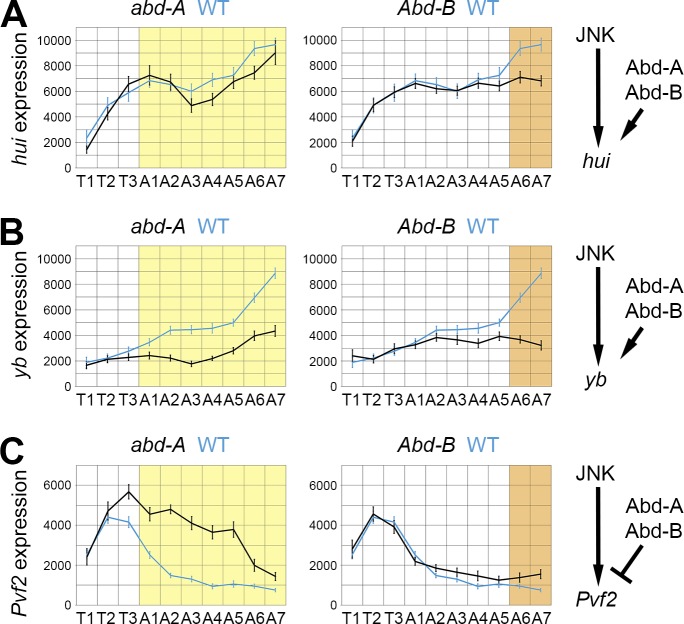
HOX regulation of other JNK target genes. Quantification of the expression (fluorescent intensity; a.u. +/- s.e.m.; n = 15) of *hui* (**A**), *yb* (**B**) or *Pvf2* (**C**) in *abd-A* (black curves; left panels) and *Abd-B* (black curves; right panels) mutant embryos, compared to WT embryos (blue curve). The expression domains of *abd-A* (yellow) and *Abd-B* (orange) are indicated in colour. Depending on the gene, Abd-A and Abd-B behave as activators or repressors of LE gene expression (right illustrations).

To determine whether HOX genes have a more general role on JNK target genes, we investigated the profiles of *hui*, *yb* and *Pvf2* genes, which like *scaf* display a complex pattern of expression along the LE. *hui* and *yb* exhibit a stronger expression in the posterior part of the embryo (‘posterior’ group), whereas *Pvf2* expression is limited to the thoracic segments (‘anterior’ group; see Fig 3B). In *abd-A* and *Abd-B* mutants, the expression of *hui* and *yb* is diminished in the respective HOX domains, indicating that both genes activate the expression of *hui* and *yb*, which is responsible for their stronger expression at the posterior pole of the embryo (Fig 6A and 6B). For *Pvf2*, we observed a strong expression increase from T3 to A6 segments in the *abd-A* mutant and a weak up-regulation in the A6/A7 segments in the *Abd-B* mutant (Fig 6C). Thus, the restricted expression of *Pvf2* in the thoracic segments is due to the inhibitory action of the two abdominal genes, especially *abd-A*. These results indicate that *abd-A* and *Abd-B* can either act as repressors or activators to shape the asymmetric expression profiles of JNK target genes in the LE.

### *abd-A* and *Abd-B* control the posterior closure of the embryo by regulating the zipping process

**Fig 7:**
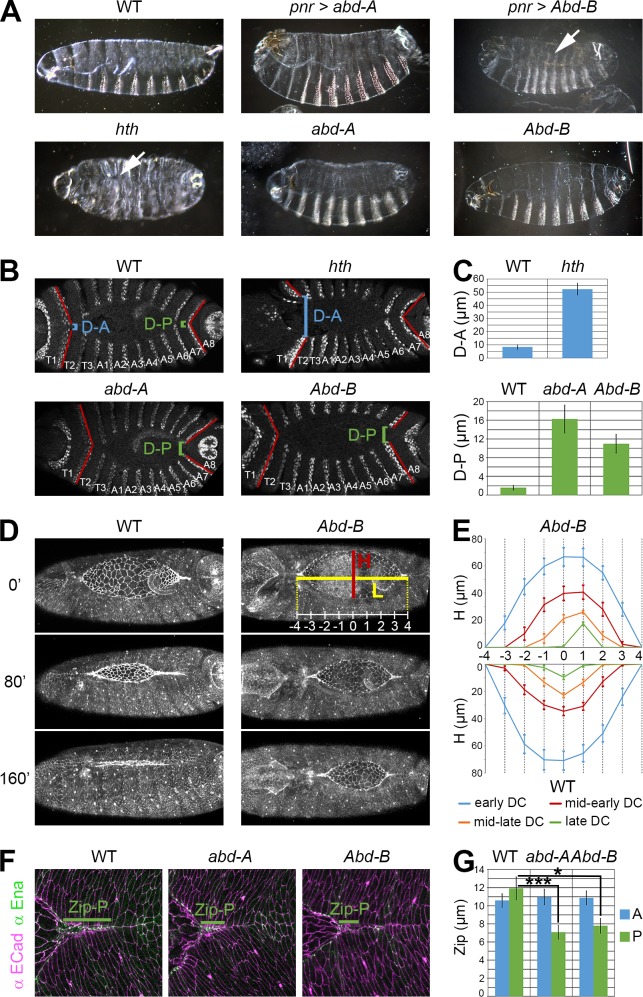
DC phenotypes of HOX mutant embryos due to zipping defects. **A)** Cuticles of a WT embryo (top left), *abd-A*- and *Abd-B*-overexpressing embryos with *pnr-GAL4* (top middle and top right, respectively), *hth*, *abd-A* and *Abd-B* mutants (bottom panels, as indicated). The overexpression of *Abd-B* induces holes in the cuticles (arrow; 48% of the dead embryos, n = 200), revealing strong closure defects. The *hth* mutant also produces a DC phenotype, with holes located in the anterior part (arrow; 76% of the dead embryos, n = 117). In contrast, the cuticles of *abd-A*-overexpressing embryos, *abd-A* and *Abd-B* mutants resemble that of WT embryos. **B)** Anti-En immunostaining of the *hth* (top right), *abd-A* (bottom left) and *Abd-B* (bottom right) mutants compared to WT embryos (top left). For *hth*, the distance in the anterior (D-A) between the two opposing En-expressing compartments of T1 is determined when the A8 segment just closed. For *abd-A* and *Abd-B*, the distance in the posterior (D-P) between the two opposing En-expressing compartments of A7 is determined when the T1 segment just closed. The red lines delimitate the posterior boundaries of the T1 and A7 segments. **C)** Quantification of D-A (μm +/- s.e.m., top panel) for the *hth* mutant and D-P (μm +/- s.e.m., bottom panel) for *abd-A* and *Abd-B* mutants. In WT embryos, T1 is almost closed (D-A = 8.3 μm +/- 1.6, n = 20) when A8 just closed, but the *hth* mutant shows a strong anterior opening ((D-A = 52.3 μm +/- 4.5, n = 17). In the posterior part of WT embryos, A7 is usually closed (D-P = 1.6 μm +/- 0.5, n = 18) when T1 just closed. In contrast, the *abd-A* and *Abd-B* mutants exhibit a posterior delay of closure (D-P = 16.3 μm +/- 3.0, n = 19, and D-P = 11.0 μm +/- 2.0, n = 16, respectively). **D)** Still images of *arm-GFP* and *arm-GFP;Abd-B* live embryos from S1 Movie. Compared to the dynamics of the *arm-GFP* (WT) embryo (left images), the closure of the *Abd-B* mutant (right images) is slowed down: at 160 minutes, the *abd-B* mutant embryo is still undergoing closure while the WT embryo is closed. Height (H) between the two opposing LE and length (L) of the projected LE at the midline between the two zipping zones that are used in C) are depicted in the top right panel. H is quantified at fixed positions along L, as indicated. **E)** Height (H, μm +/- s.e.m.), as defined above, during early DC (seam < 25%; blue), mid-early DC (25% < seam > 50%; red), mid- late DC (50% < seam > 75%; orange), and late DC (seam > 75%; green) at fixed positions of L. Whereas the control embryos (n = 9) close right in the middle, *Abd-B* mutants (n = 6) exhibit a posterior shift of their closure point at the end of DC (shift corresponding to approximately 1 segment). **F)** Anti-Ena (green) and anti-ECad (magenta) immunostainings showing the posterior zipping zones (Zip-P) in the A6 segment of a WT embryo (left) and of *abd-A* (middle) and *Abd-B* (right) mutants. The zipping zone is defined by the area where the two opposing LE are in close contact till the formation of a stable adherens junction (stained with ECad). **G)** Quantification of the anterior (A of the T2 or T3 segment, blue) and posterior (P of the A6 or A7 segment, green) zipping zones of the WT embryo and the two HOX mutants. Whereas no variation is observed in the anterior part (no statistical significance), the Zip-P of *abd-A* (7.1 μm +/- 0.7) and *Abd-B* (7.8 μm +/- 0.8) are reduced compared to the WT embryo (11.9 μm +/- 1.2). Multiple comparisons of Zip-A or Zip-P between WT, *abd-A* and *Abd-B* were performed using the Dunnett test with R (*: p < 0.05; **: p < 0.01; ***: p < 0.001). For Zip-A and Zip-P of WT embryos, n = 15; for *abd-A*, n(Zip-A) = 15 and n(Zip-P) = 16; for *Abd-B*, n(Zip-A) = 10 and n(Zip-P) = 13.

In order to assess the functional relevance of Hox gene regulation during DC, we analysed the phenotype of HOX gain- and loss-of-function embryos. Overexpression of *Abd-B* in the dorsal ectoderm results in a strong DC phenotype, as revealed by holes present in the embryonic dorsal cuticles (Fig 7A). Therefore, ‘posteriorisation’ of the LE through Abd-B overexpression, which leads to ectopic activation of JNK target genes (Fig 5E and 5F), is detrimental to DC, indicating the importance of proper patterning of the LE along the AP axis. In contrast, *abd-A* overexpression does not create cuticle holes (Fig 7A), which might be due to the regulation of a different set of target genes compared to *Abd-B*. To characterize the role of the HOX genes in their normal domain of expression, we analysed the effect of their loss-of-function. First, we observed the cuticles of the *abd-A* and *Abd-B* mutants, which resemble that of WT embryos (Fig 7A). The *hth* mutant exhibits a strong DC phenotype, with holes located in the anterior part. This regionalisation of the phenotype is likely reflecting the higher expression of *hth* in the thoracic part of the embryo [45]. In addition, this strong phenotype is probably due to the effect on several HOX genes, in particular the thoracic ones (*Scr*, *Antp* and *Ubx*), as previously shown (Fig 5G). We then decided to analyse in more details the DC phenotype of the HOX genes by analysing segment closure during early DC. In the WT embryo, closure of the posterior segment A8 and closure of the anterior segment T1 take place approximately at the same time (Fig 7B). In the *hth* mutant, when A8 has just closed, the segment T1 is far from being closed, revealing a strong delay in the closure of the anterior part of the embryo (Fig 7B and 7C). We also analysed the *abd-A* and *Abd-B* mutants, and both show a delay in the closure of the posterior segment A8 (Fig 7B and 7C). Therefore loss of HOX gene function triggers DC defects in their respective field of action, and the severity of the phenotype depends on the number of HOX genes affected.

To get better insight into the role of individual HOX genes on the dynamics of DC, we performed live imaging on *Abd-B* mutant embryos, which display a clear delay in DC (Fig 7D; S1 Movie). Despite this delay, they eventually close and show no hole in their dorsal cuticles (Fig 7A), reflecting the high degree of adaptability and robustness of DC in challenged conditions [8, 46, 47]. Analysis of the point of closure at the LE indicated that closure of the *Abd-B* mutant was shifted towards the posterior, compared to WT embryos whose final point of closure is located at the middle of the LE (Fig 7E). Early delay observed in fixed embryos (Fig 7B and 7C) is thus maintained throughout DC to trigger an asymmetric closure, indicating that no compensatory mechanism takes place. The posterior shift and closure delay seen in *Abd-B* embryos can be explained by a progressive reduction of the posterior speed of closure. Indeed, this speed shows a clear reduction of 59% (mid DC) and 78% (late DC) (S5A Fig). The anterior speed also decreases, but to a less extent (58% in late DC; see Discussion).

The DC defect of the *hth* mutant seems to be generated by an inefficient zipping in the anterior part, but the strong phenotype makes it difficult to interpret. We therefore analysed the zipping zone of the *abd-A* and *Abd-B* mutant embryos. Antibody stainings indicated that the establishment and positioning of LE markers and of the zipping zone are correct, such as the ANC-localised protein Enabled (Ena), the adherens junction protein ECad and the septate junction protein Dlg (Figs 7F and S5B). However we noticed a reduction in the length of the posterior zipping zone in both mutants (Fig 7F and 7G; S5B and S5C Fig). The WT zipping zone, in the anterior or posterior part, is about 11 μm long on average, which corresponds to the length previously published [6]. Whereas the anterior zipping zone of the posterior HOX mutants does not vary, there is a reduction of the posterior one (between 7 and 8 μm). As the zipping zone provides a force that is important for DC [6, 8, 11], reduction of its length is likely to affect the dynamics of closure, as observed with the *abd-A* and *Abd-B* mutants. We finally tested whether the action of the HOX genes takes place in the dorsal most cells of the ectoderm. Reducing *Abd-B* expression by RNAi (*Abd-B*^*i*^) with *pnr-GAL4* (expression in the dorsal ectoderm and amnioserosa) induces a phenotype of posterior closure delay, whereas the *AS-GAL4* driver (expression in the amnioserosa only) does not give any DC phenotype (S5D and S5E Fig). This result indicates that the activity of *Abd-B* in the dorsal most cells, and not in the amnioserosa, is required to control DC. Altogether, our results indicate that the HOX genes are essential for the correct timing of closure of the segments of the *Drosophila* embryo by influencing the formation of the zipping zone and thus the efficacy of zipping. They further demonstrate the importance of the interplay between the HOX genes and the JNK pathway in the LE to control the dynamics of DC along the AP axis.

## Discussion

Our identification of several new JNK target genes during DC and analysis of their quantitative expression pattern uncover the complex transcriptional response taking place in the LE morphogenetic domain. Results reveal an intricate regulatory network integrating multiple signalling layers. In this process, AP positional information and JNK signalling cooperate to generate a highly patterned, yet apparently smooth and regular LE. Mutant analysis shows that LE partitioning into discrete domains is important to control the coordination, hence dynamics of the whole closure process.

The LE is a major component of DC, being the site of JNK activity and actin cable assembly; it also provides an active boundary with the amnioserosa, driving epidermal spreading and seamless tissue sealing. Therefore, it is important to determine its morphogenetic and signalling features and how these are dynamically controlled. To this end, first we identified a new set of target genes whose expression in the dorsal ectoderm is dependent on JNK activity during DC. Transcriptome analysis allowed us to identify 1648 independent genes which are up- or down-regulated in JNK activated embryos. Filtering of this large set led us to focus on a group of 194 genes whose expression was analysed by quantitative *in situ* hybridization in different genetic conditions. Transcriptional profiling unveiled 31 *Drosophila* JNK target genes of which only a fraction was already known, including *jra/jun*, *reaper*, *Zasp52* and *scab* [21, 30, 31]. Amongst novel targets were also *Scaf* and *Rab30* the role of which we have previously described during DC [17, 22]. Two categories of JNK target genes can be distinguished: genes that are specifically expressed in the LE and genes whose expression is more ubiquitous in the dorsal ectoderm. Genes belonging to the latter category may play a general role in the ectoderm under the control of different pathways, for example in the case of *Rab30*. In contrast, LE-specific genes likely play a specific role during DC, as is the case for *puc*, *dpp* and *scaf*. However, it is also possible that some of the new genes, despite being expressed in the embryo in a JNK-dependent manner, are not involved in DC. These target genes thus remain under the control of JNK, but are functionally ‘silent’ during DC. This behaviour is best illustrated by the *reaper* gene, whose expression is JNK-dependent in the embryo (Fig 1B and 1C), but which does not seem to have any function in the LE, only later during development or at the adult stage.

Surprisingly, quantitative analysis of LE-specific gene expression profiles showed a variety of previously uncharacterized expression patterns along the LE, with two levels of regulation, AP and segmental. These observations reveal a new property of the LE which appears highly patterned along the AP axis, contrasting with the homogenous and linear structure previously envisioned. In addition, the higher order regulation that emerges from these results provides every LE cell with its own identity through the cross-talk between JNK, AP and segmental information. Such cell-level patterning through signalling crosstalk [48] is likely essential for coordination and robustness of closure as well as segment matching. In this view, recent work showed that Wg and JNK interact at the LE to control the formation of specific mixer cells at segment boundaries [49].

Previous work showed that, instead of acting independently, HOX and segmentation genes can be coupled to regulate target genes in the embryo [50]. Here, we reveal an additional layer of regulation involving the ‘morphogenetic’ JNK signalling pathway. During DC, JNK acts as a tissue-specific switch whose activity can be regulated by HOX and segmentation pathways, providing positional information and segmental organization to a moving tissue boundary. Thus, a multi-layered or ‘onion-like’ regulatory model allows for several levels of regulation/information to pile up in order to regulate individual cellular behaviours important for tissue morphogenesis. Each layer can act positively or negatively on LE target gene expression, generating a complex repertoire of regulatory pathways. Distinct categories of expression profiles have been identified in this study through the analysis of individual target genes, with likely more gene-specific patterns to be anticipated. For example, the same HOX gene (*abd-A* or *Abd-B*) can have activating or repressive activity according to the target gene, as is the case for the transcription factor En [39]. Molecular functional characterization of cis-regulatory elements controlling LE gene expression will bring a more detailed view of how transcription factor complexes are formed, how specificity of DNA recognition is achieved and how activating or repressive activities are regulated to generate LE patterning.

*scaf* proves to be a remarkable case among the JNK target genes, summarizing the different levels of regulation that can be integrated into a single promoter. Not only is it strongly expressed in the LE in a JNK-dependent manner, but it is also regulated by both the segmentation gene *en* and the HOX genes. In particular *scaf* displays a transcriptional response induced by all the trunk HOX genes tested, being positively controlled by *Scr*, *Antp*, *Ubx* and *Abd-B* and negatively by *abd-A*. It can therefore be considered as a general HOX target gene, i.e. regulated by most Hox paralogs, as previously defined [40]. Another example of a general target is the *Drosophila* gene *optix*, which is activated by the head HOX genes *labial* and *Deformed* (*Dfd*) and inhibited by the trunk HOX genes [51]. Nonetheless the general HOX target genes do not represent the majority. A genomic analysis in the *Drosophila* embryo identified more than 1500 genes regulated by at least one of the six HOX paralogs tested (*Dfd*, *Scr*, *Antp*, *Ubx*, *abd-A*, *Abd-B*) [52]. Only 1.3% of these genes are regulated by the six paralogs and 1.5% by the five paralogs that we used in our study. Interestingly more than 40% of the ~1500 HOX target genes are also present in the JNK genomic data set that we obtained. This strong overlap well reflects the fact that the LE runs along most of the body AP axis encompassing the thorax and abdomen. More importantly, it also indicates that AP patterning plays a crucial role in the regulation of DC, as shown in this study.

Live imaging and mathematical modelling revealed asymmetries in the geometry and zipping process along the AP axis [8, 53], which can be attributed to local constraints induced by head involution and apoptosis [10, 26]. Head involution is concomitant with DC and induces tension in the anterior part of the embryo, explaining why the DC phenotypes are almost exclusively observed in the anterior part, leading to the so-called ‘anterior-open phenotype’. The exception to this rule is the experimental manipulation of the posterior zipping rate through localized laser ablation of the amnioserosa close to the canthus, which induces a strong delay of posterior closure [26]. Our results with the *abd-A* and *Abd-B* mutants show that posterior delay can also be obtained in genetically-perturbed embryos. However, while anterior zipping is slightly up-regulated when posterior zipping is laser-targeted [26], we showed that the anterior speed of closure is diminished in the *Abd-B* embryo. Thus, compensatory mechanisms may only appear when tissue integrity is severely impaired. Apoptosis was also proposed to participate in the asymmetric properties of DC [10]. Delamination of apoptotic cells in the anterior amnioserosa produces forces that are responsible for a higher rate of anterior zipping. However, the phenotype that we observed with the *abd-A* or *Abd-B* mutation cannot be attributed to defects in this mechanism, as the rate of apoptosis is already very low in the posterior amnioserosa. Therefore, our data reveal a genetic control of zipping through precise transcriptional regulation in the LE. Overall, our work provides a framework for apprehending how the HOX selector genes and their cofactors collaborate with other signalling pathways to generate specific transcriptional responses allowing morphogenetic patterning and proper coordinated development.

## Materials and methods

### Fly stocks

The following fly stocks were used in this study: *w*^*1118*^ (BDSC#3601) as WT flies, *y w hep*^*1*^ and *y w hep*^*r75*^ [1], *UAS-hep*^*act*^ (BDSC#9306), *puc*^*E69*^ (*puc-Z*)[54], *69B-GAL4* (BDSC#1774), *pnr-GAL4* (BDSC#3039), *en-GAL4* (gift from A. Brand), *AS-GAL4* (*c381-GAL4*; BDSC#3734), *en*^*X31*^ [55], *UAS-en* [56], *UAS-HA*::*VP16*::*en* [39], *Scr*^*17*^ (BDSC#3400), *Antp*^*[Ns-rvC4]*^ (BDSC#1830), *Ubx*^*1*^ (BDSC#626), *abdA*^*M1*^ and *AbdB*^*D18*^ [57], *UAS-Scr*::*HA*, *UAS-Ubx*::*HA* and *UAS-abdA*::*HA* [58], *UAS-Antp* [59], *UAS-AbdBm* (BDSC#913), *UAS-AbdB*^*i*^ (VDRC#12024)[60], *UAS-Dicer2* (BDSC#24650), *hth*^*P2*^ [61], *TM3*,*dfd-lacZ* [62]. For live imaging, we used *arm*::*GFP* (BDSC#8556 on chromosome II; BDSC#8555 on chromosome III) and created the recombinant line *arm*::*GFP*,*Abd-B*^*D18*^ that we balanced with *TM3*,*twi-GAL4*,*UAS-GFP* (BDSC#6663) to select the homozygote mutant embryos. We also created the following line for the *Abd-B* RNAi: UAS-*Dicer2;UAS-CD8*::*RFP*,*UAS-Abd-B*^*i*^, allowing the selection of *AbdB*^*i*^ embryos thanks to the expression of CD8::RFP.

### Microarray screen

Three genetic conditions were compared: wild-type (WT, *w*^*1118*^), gain of function (GOF, *69B-GAL4 > hep*^*act*^) and loss of function (LOF, *hep*^*1*^*/hep*^*r75*^). The *69B-GAL4* driver allows a uniform expression in the ectoderm. For the LOF embryos, we crossed *y w hep*^*r75*^*/FM6* virgin females to *y w hep*^*1*^*/Y* males to obtain *y w hep^r75^/y w hep^1^* virgin females that were then crossed to *y w hep*^*1*^*/Y* males. 100% of the embryos coming from this last cross are mutant for DJNKK because *hep*^*1*^ behaves as a total loss of maternal function while being zygotically viable [1]. Two hour egg collections were incubated at 25°C during the time necessary to obtain 85 to 90% embryos undergoing DC (~9 hrs). Because of a certain variability of development that we were not able to control, a fraction of each embryo was systematically tested by *dpp in situ* hybridization, and the collections that did not correspond to the definite criterion (above 85% of stage 13–14 embryos) were eliminated. Embryos from three biological replicates for each genetic condition were dechorionated with 50% bleach, frozen in nitrogen liquid and stored at -80C. Embryos were homogenized in RLT buffer (QIAGEN)/beta-mercaptoethanol using a conventional rotor–stator homogenizer. Total RNA was prepared with the Qiagen RNeasy mini kit for animal tissues according to the manufacturer's instructions. Biotinylated cRNA were prepared according to the standard Affymetrix protocol from 2 μg total RNA. The biotinylation, the hybridization and the scan were done at the Affymetrix Platform located at the Institut Curie, Paris. Bioinformatics analysis was performed using RMA ("Robust Multi-array Average") and SAM ("Significance Analysis Microarray) under R/Bioconductor. The false discovery rate (FDR) of the GOF analysis is excellent (0.85%), evaluating the number of false positive to 14 out of the 1648 genes identified. The FDR associated with the LOF comparison is 3.19%, i.e. corresponding to 3 or 4 false positive out of the 113 isolated genes. The enrichment scores were obtained using the DAVID Bioinformatics Resources [27]. Comparison of microarray data sets was performed with THEA [29]. Microarray data have been deposited in the Gene Expression Omnibus (accession number GSE21805).

### *In situ* hybridizations

The embryos were prepared using a standard protocol. Briefly, embryos resulting from 16hr collections at 25°C, or 29°C for the overexpression experiments, were dechorionated in 50% bleach, fixed in 4% formaldehyde and devitellinized in a heptane:methanol (1:1) mix. Embryos were then freshly used or kept at -20°C for a few weeks. Digoxigenin (DIG)-labelled RNA probe synthesis was performed with T3, T7 or Sp6 RNA polymerases as recommended (Promega, New England Biolabs) from cDNA collections (Drosophila Genomics Resource Center). Classical *in situ* hybridization (ISH) experiments were done using a standard protocol with the anti-DIG antibody conjugated to alkaline phosphatase (1/2000; Roche Diagnostics). Staining was performed with NBT/BCIP reagent (Sigma). For fluorescent ISH coupled to immuno-fluorescence (FISH-IF), the second fixation step was accomplished using freshly prepared paraformaldehyde and the proteinase K treatment was omitted. Primary antibodies were: rabbit anti-En (1/100; Santa Cruz), mouse anti-Scr (1/50; DSHB), mouse anti-Antp (1/50; DSHB), mouse anti-Ubx (1/50; DSHB), mouse anti-abd-A (1/100; DSHB), mouse anti-Abd-B (1/50; DSHB), mouse anti-HA (1/500; Covance), chicken anti-β Galactosidase (1/500; GeneTex). Sheep anti-DIG antibody coupled to horseradish peroxidase (1/500; Roche Diagnostics) was joined to the secondary antibodies: anti-rabbit Al488 (1/400; Molecular Probe), anti-mouse Cy5 (1/100; Jackson ImmunoResearch), anti-chicken DyLight649 (1/200; Life Technologies). Revelation was done twice for 5–10 minutes with Tyramide Signal Amplification (PerkinElmer). Embryos were mounted in Mowiol 4–88 Reagent (Calbiochem). Images were acquired with a LSM 710 Zeiss confocal microscope using a 40X Objective.

### mRNA signal quantification at the LE

Confocal Z sections that entirely encompass the LE of 15 to 20 embryos per genotype (except for the *en* mutant: n = 10) were acquired as 2048X2048–12 bit images and maximum intensity projections were created. To quantify the fluorescence signal in the LE, we developed CurvedPeriodicity, a standalone user-friendly program coded in Matlab (the Mathworks). The steps of the image pre-treatment are 1) to correct the background measured in the amnioserosa to linearize the intensity signal to fluorescence, 2) to extract the ribbon containing the LE and to make it linear, 3) to split the ribbon by semi-automatic demarcation of the segment boundaries, and 4) to normalize each segment in length. From these data, the software quantifies the mRNA signal as the mean intensity projections along the LE. It yields both the signal distribution per segment along the LE (*d*_*LE*_) and the mean distribution along a Normalized Segment (*d*_*NS*_). Segments are composed, on average, of 10 cells with 7 in the anterior compartment and 3 in the posterior compartment. Therefore *d*_*NS*_ was divided in 10 regions and the parasegmental boundary was set between the 7^th^ and 8^th^ cells. CurvedPeriodicity calculates the average signal from each cell of each segment, and also the AP ratio per segment. Taking advantage of the normalization, the software is able to pool the confocal images per condition, synthetizing the data and extracting the mean and s.d. for *d*_*LE*_ and *d*_*NS*_, and to export it directly into an Excel file. In case of negative numbers (i.e. expression below background), the expression level was set to 0. For the AP analysis, the mean of the signals from each segment from T1 to A7 (n embryos; n = 15 to 20) was calculated. For the compartmental analysis, the signals for a given cell of all the segments (n segments = 150 to 200, with 10 segments per embryo) were averaged.

### Quantitative PCR

We used the same RNA samples (control: *w*^*1118*^, GOF: *69B-GAL4 > hep*^*act*^, LOF: *hep*^*1*^*/hep*^*r75*^) than the ones prepared for the microarray analysis. Reverse-transcription was performed with SuperScript III Reverse Transcriptase (Invitrogen Life Technology) after DNase I digestion with a mix of oligo-dT and random primers. Q-PCR was performed with the Mastermix Plus for SYBR Green containing Rox (Eurogentec) with the endogenous *rp49* gene for normalization. The list of primers that were used is available upon request. Standard curves of all the couples of primers presented an efficacy of amplification comprised between 95% and 110% with a coefficient of determination R^2^ of at least 0.995. For each condition we did three technical replicates from one biological sample. Results were analysed with the StepOne software v2.1 (Applied Biosystems).

### Cuticle preparations

Embryos were collected for 24 hours and incubated at 25°C or 29°C to let the wild-type larvae crawl away. The cuticles of unhatched (dead) embryos were prepared as following: embryos were dechorionated, mounted in 100 μl of a mix (1:1) made of lactic acid and Hoyer’s mounting medium (30 g of gum Arabic, 50 ml of distilled water, 200 g of chloral hydrate, 20 ml of glycerol), and incubated overnight at 65°C. Images were taken with a Nikon Coolpix 990 camera under a Leica DMR microscope.

### Immunostaining and quantification

Embryos resulting from 16hr collections at 25°C were dechorionated in 50% bleach, fixed in 4% formaldehyde and devitellinized in a heptane:methanol (1:1) mix. Embryos were then quickly rinsed with PBS-triton X-100 0.1% and the following primary antibodies were then used: rabbit anti-En (1/200; Santa Cruz), mouse anti-Ena (1/100, DSHB), rat anti-ECad (1/100, DSHB) and/or mouse anti-Dlg (1/200, DSHB). After washes, secondary antibodies were added: anti-rabbit Al488 (1/400; Molecular Probe), anti-mouse Al488 (1/400; Molecular Probe), anti-mouse Al546 (1/400; Molecular Probe), anti-rat Al546 (1/400; Molecular Probe) and/or anti-rat Al647 (1/400; Jackson ImmunoResearch). After washes, embryos were mounted in Mowiol 4–88 Reagent (Calbiochem). Images were acquired with a LSM 780 Zeiss confocal microscope using a 25X or 63X Objective.

Closure delay was quantified by measuring with ImageJ the distance between the matching posterior compartments (stained with En) of the T1 (D-A) or A7 (D-P) segment when the opposing segment (A8 or T1, respectively) just closed. For the zipping zone, quantification was done with ImageJ by tracing a line from the point where the two opposing LE are in close contact to the point where a stable junction (adherens junction with ECad or septate junction with Dlg) is formed.

### Live imaging and quantification

Embryos collected from overnight egg-laying were dechorionated (50% bleach) and stage 13 embryos (at the onset of DC) were selected under the binocular. They were mounted in Halocarbon 95 oil between two cover slips separated by spacers, glued on their ventral part. Two hydrating chambers (watered cotton) were positioned on the sides. 1024X1024 Z-stacks (1 to 2 μm/image) were acquired over 4 hours with a Zeiss 780 confocal microscope. Distances necessary for calculation of the speed of closure, H and L (Fig 7) were measured from maximum intensity projections of optimized Z sections with FIJI. Stages of DC (expressed as % of DC) were estimated by calculating the ratio of the seam (fused LE) and L (projected LE at the midline). L was divided in 8 sub-domains (corresponding approximately to 8 segments), as previously described [63].

## Supporting information

S1 Fig Microarray analysis of the JNK response during DC. **A)** *In situ* hybridizations of stage 13 embryos showing *dpp* expression in the three conditions used to prepare the RNAs for microarray analysis: WT (*w*^*1118*^; top), GOF (*69B-GAL4 > hep*^*act*^; middle) and LOF (*hep*^*1*^*/hep*^*r75*^; bottom). Activation of the JNK pathway in the ectoderm with *69B-GAL4* leads to a lateral expansion of *dpp* expression (dotted area), whereas in the *hep*^*1*^*/hep*^*r75*^ mutant, *dpp* expression is lost specifically in the LE, but not in the other tissues. **B)** Distribution of the number of genes that are up-regulated (green) and down-regulated (red) by the JNK pathway (with a fold change (FC) superior to 1.5 or inferior to -1.5) in the GOF (top) and LOF conditions (bottom).
(TIF)

S2 Fig Anterior-posterior expression of the twelve LE genes. FISH-IF (first and third columns) and mRNA signal quantifications at the LE (second and fourth columns) are shown for each LE gene. mRNA staining is in red and anti-En immuno-fluorescence is in green. Bar diagram: average mRNA signal intensities at the LE in each segment, expressed as fluorescent intensity (a.u. +/- s.e.m.). This figure is a supplement of Fig 3B.
(TIF)

S3 Fig Segmental expression of the twelve LE genes. FISH-IF (columns 1, 2, 4 and 5) and mRNA signal quantifications at the LE (columns 3 and 6) are shown for each LE gene. mRNA staining is shown in red alone (columns 1 and 4) or merged with anti-En immuno-staining (in green)(columns 2 and 5). Bar diagram: average mRNA signal intensities at the LE in the ten cells of the segments, expressed as fluorescent intensity (a.u. +/- s.e.m.). This figure is a supplement of Fig 3C.
(TIF)

S4 Fig Regulation of *scaf* expression by *en* and the HOX genes. **A)** Quantification of *scaf* expression (expressed as fluorescent intensity; a.u. +/- s.e.m.) in the LE of *en* mutant embryos (n = 10, FISH-IF shown in Fig 4A). The anterior limit for quantification corresponds to the dorsal ridge abutting the head segments, whereas the posterior limit was set just anteriorly to the place where *scaf* expression starts to expand in the more lateral epidermis (see Fig 4A). The LE was then divided in 10 equal segments for quantification with CurvedPeriodicity. Top panel: quantification in the A (blue) and P (green) compartments along the AP axis. Bottom panel: quantification in the A and P compartments of segments A1 to A6 of the *en* mutant, compared to WT embryos, showing the absence of negative regulation on *scaf* expression in the P compartments. **B)** FISH-IF showing *scaf* expression (*scaf* mRNA is in red either merged or not with anti-En staining in green) in the *abd-A* mutant (quantification shown in Fig 5A). **C)** FISH-IF in embryos overexpressing *abd-A* with *pnr-GAL4* (quantification shown in Fig 5B). **D)** *scaf* expression in *Scr* (first column), *Antp* (second column), *Ubx* (third column) and *Abd-B* (fourth column) mutants. First row: quantification of *scaf* expression (expressed as fluorescent intensity; a.u. +/- s.e.m., n = 15 for each mutant) in each compartment (anterior: blue; posterior: green) of each segment along the AP axis. Each main expression domain of the corresponding HOX gene is indicated by a color: grey for *Scr*, pink for *Antp*, blue for *Ubx* and orange for *Abd-B*. Second row: FISH-IF showing *scaf* mRNA (red). Third row: FISH-IF showing *scaf* mRNA (red) with anti-En staining (green). This figure is a supplement of Fig 5D. **E)** FISH-IF showing *scaf* expression in the *hth*^*P2*^ mutant (*scaf* mRNA in red, anti-En staining in green) (quantification shown in Fig 5G).
(TIF)

S5 Fig *abd-A* and *Abd-B* posterior closure defects. **A)** Speed of closure (nm/s +/- s.e.m.) in the anterior and posterior regions of control (pink; n = 9) and *Abd-B* mutant (purple; n = 6) embryos during early DC (seam < 33%), mid DC (33% < seam > 66%) and late DC (seam > 66%). A strong reduction of the posterior closure speed was observed from mid DC (59%) to late DC (78%) in *Abd-B* embryos. The anterior speed also decreased, especially during late DC (58%). This panel is a supplement of Fig 7D and 7E. **B)** and **C)** Dlg-defined zipping zones of WT, *abd-A* and *Abd-B* embryos. **B)** Anti-Dlg (red) immunostainings showing the posterior zipping zones (Zip-P) in the A6 segment of a WT embryo (left) and of *abd-A* (middle) and *Abd-B* (right) mutants. The zipping zone is defined by the area where the two opposing LE are in close contact till the formation of a stable septate junction (marked with Dlg). **C)** Quantification of the anterior (A, blue) and posterior (P, green) zipping zones of the WT embryo and the two HOX mutants. Whereas no variation is observed in the anterior part (no statistical significance), the Zip-P of *abd-A* (7.7 μm +/- 0.5) and *Abd-B* (7.3 μm +/- 0.7) are reduced compared to WT embryos (11.2 μm +/- 1.0). As there is no homogeneity of variance for Zip-A, the Kruskal & Wallis test was performed with R, whereas multiple comparisons of Zip-P between WT, *abd-A* and *Abd-B* were performed using the Dunnett test (*: p < 0.05; **: p < 0.01; ***: p < 0.001). For WT embryos, n(Zip-A) = 7 and n(Zip-P) = 13; for *abd-A*, n(Zip-A) = 9 and n(Zip-P) = 14; for *Abd-B*, n(Zip-A) = 11 and n(Zip-P) = 15. This figure is a supplement of Fig 7F and 7G. **D)** and **E)** Inhibition of *Abd-B* expression in the dorsal ectodermal cells induces posterior DC phenotype. **D)** Anti-En (green) and anti-RFP (red) immunostainings of embryos expressing *Abd-B* RNAi (*Abd-B*^*i*^) and CD8::RFP either with *pnr-GAL4* (left panels) or with *AS-GAL4* (right panels). Whereas expression in the AS alone has no effect (when A8 closes, T1 is just about to close), expression in the dorsal ectoderm and transiently in the AS with *pnr-GAL4* triggers a delay in the posterior closure: when A8 closes, the anterior segments T1, T2 and T3 are all closed (blue arrow). **E)** Quantification of the penetrance of the posterior DC defects (%) of *pnr > Abd-B*^*i*^, *CD8*::*RFP* (n = 12) and *AS > Abd-B*^*i*^, *CD8*::*RFP* (n = 16). The Fisher’s exact test (nonparametric test for two independent samples and a binomial distribution) was used (***: p < 0.001).
(TIF)

S1 Movie *arm-GFP* (left) and *AbdB^D18^*,*arm-GFP* (right) live embryos. Frames were taken every 20 minutes.
(AVI)

S1 Table Common genes of the GOF and LOF microarray screen with their corresponding fold change (FC). (DOCX)

S2 Table Over-represented Gene Ontology (GO) terms in the list of JNK up-regulated genes in the GOF screen (using DAVID). (DOCX)

S3 Table Over-represented Gene Ontology (GO) terms in the list of JNK down-regulated genes of the GOF screen (using DAVID). (DOCX)

S4 Table Description of the 31 JNK target genes. (DOCX)
