# Supplementary figures and images for: Signalling crosstalk at the leading edge controls tissue closure dynamics in the Drosophila embryo
Source: PLoS Genet. 2017 Feb 23;13(2):e1006640. doi: 10.1371/journal.pgen.1006640 (PMC5344535; doi:10.1371/journal.pgen.1006640)

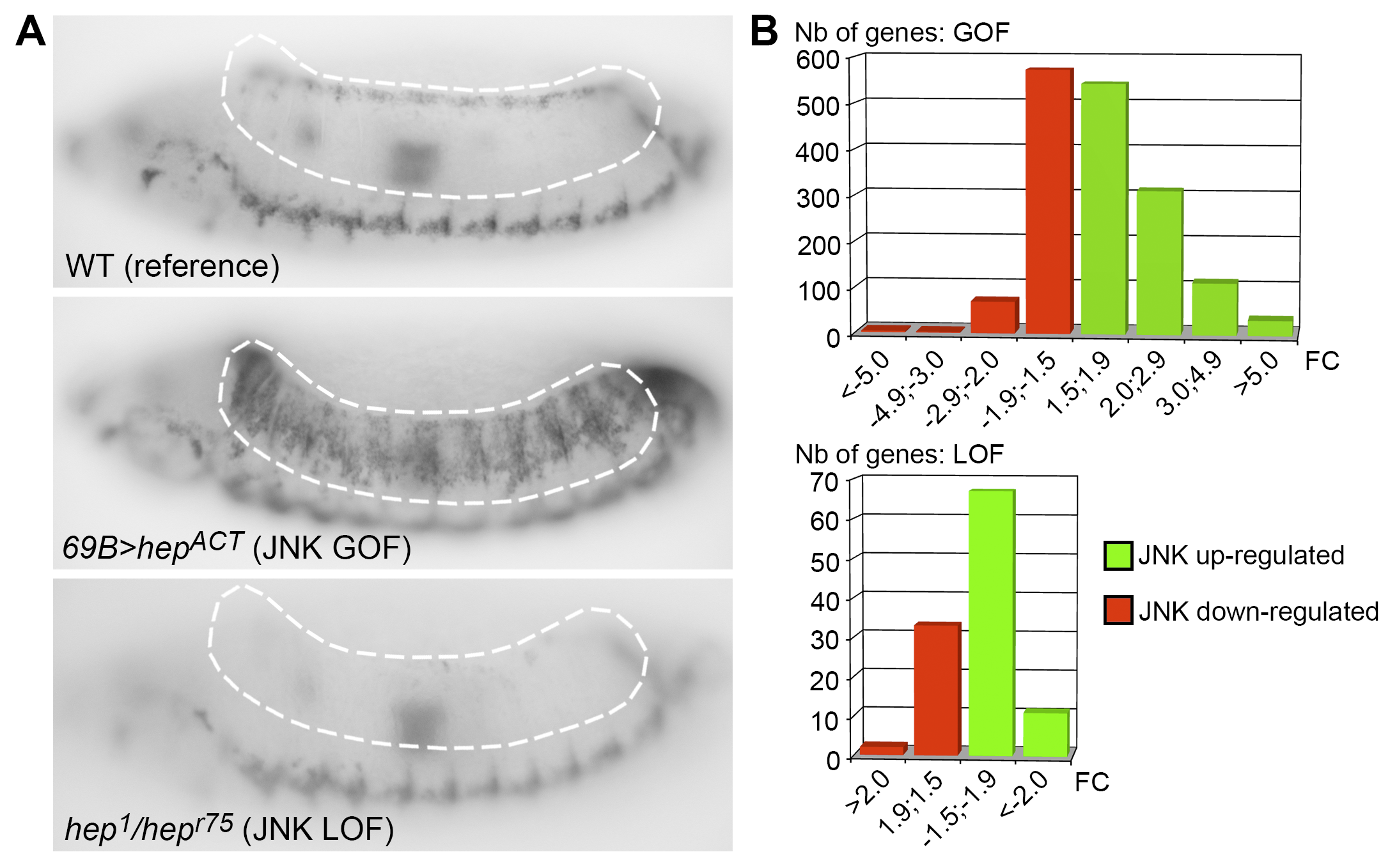

Supplement: S1 Fig — A) In situ hybridizations of stage 13 embryos showing dpp expression in the three conditions used to prepare the RNAs for microarray analysis: WT (w1118; top), GOF (69B-GAL4 > hepact; middle) and LOF (hep1/hepr75; bottom). Activation of the JNK pathway in the ectoderm with 69B-GAL4 leads to a lateral expansion of dpp expression (dotted area), whereas in the hep1/hepr75 mutant, dpp expression is lost specifically in the LE, but not in the other tissues. B) Distribution of the number of genes that are up-regulated (green) and down-regulated (red) by the JNK pathway (with a fold change (FC) superior to 1.5 or inferior to -1.5) in the GOF (top) and LOF conditions (bottom). (TIF) [file pgen.1006640.s001.tif]

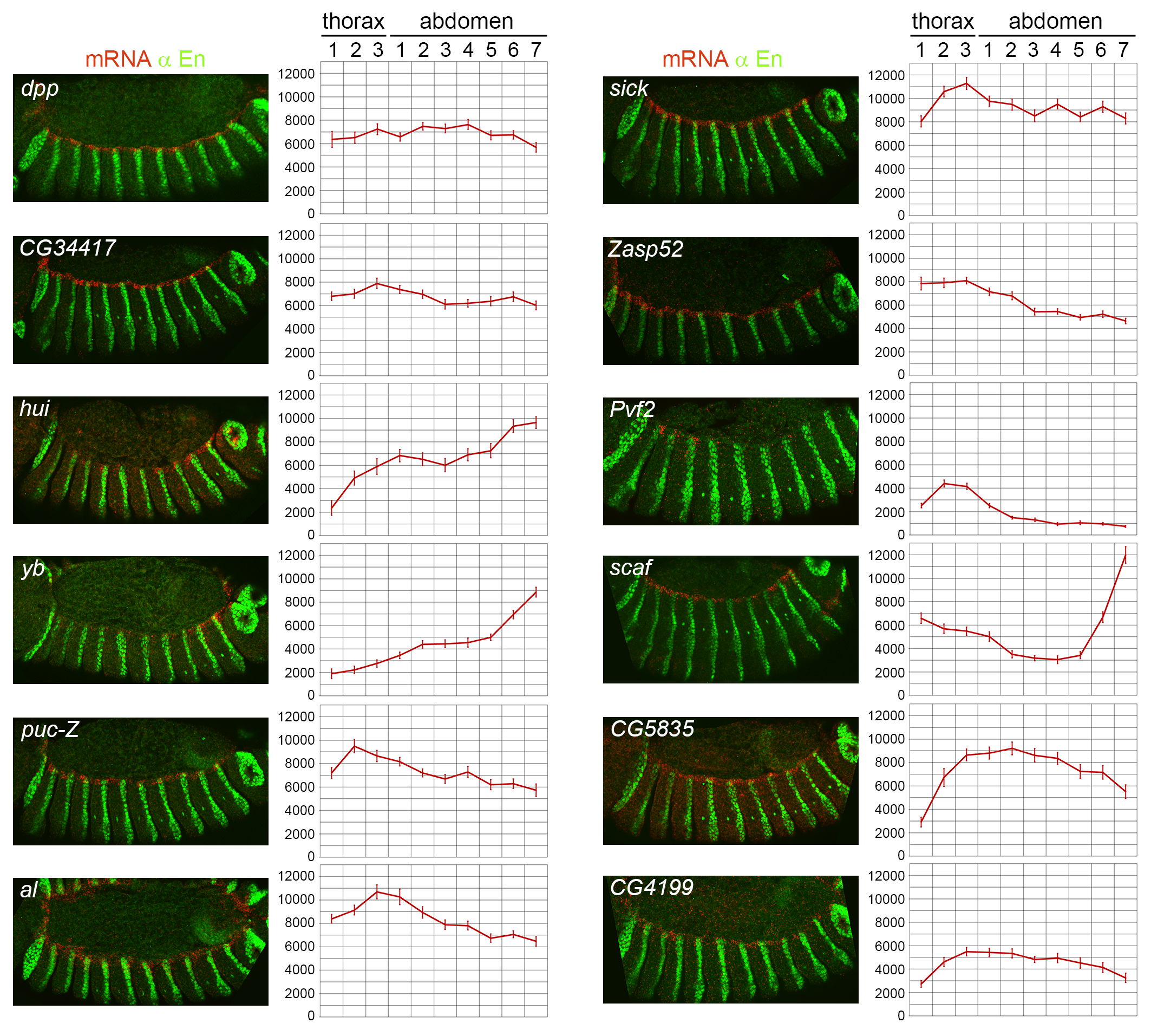

Supplement: S2 Fig — FISH-IF (first and third columns) and mRNA signal quantifications at the LE (second and fourth columns) are shown for each LE gene. mRNA staining is in red and anti-En immuno-fluorescence is in green. Bar diagram: average mRNA signal intensities at the LE in each segment, expressed as fluorescent intensity (a.u. +/- s.e.m.). This figure is a supplement of Fig 3B. (TIF) [file pgen.1006640.s002.tif]

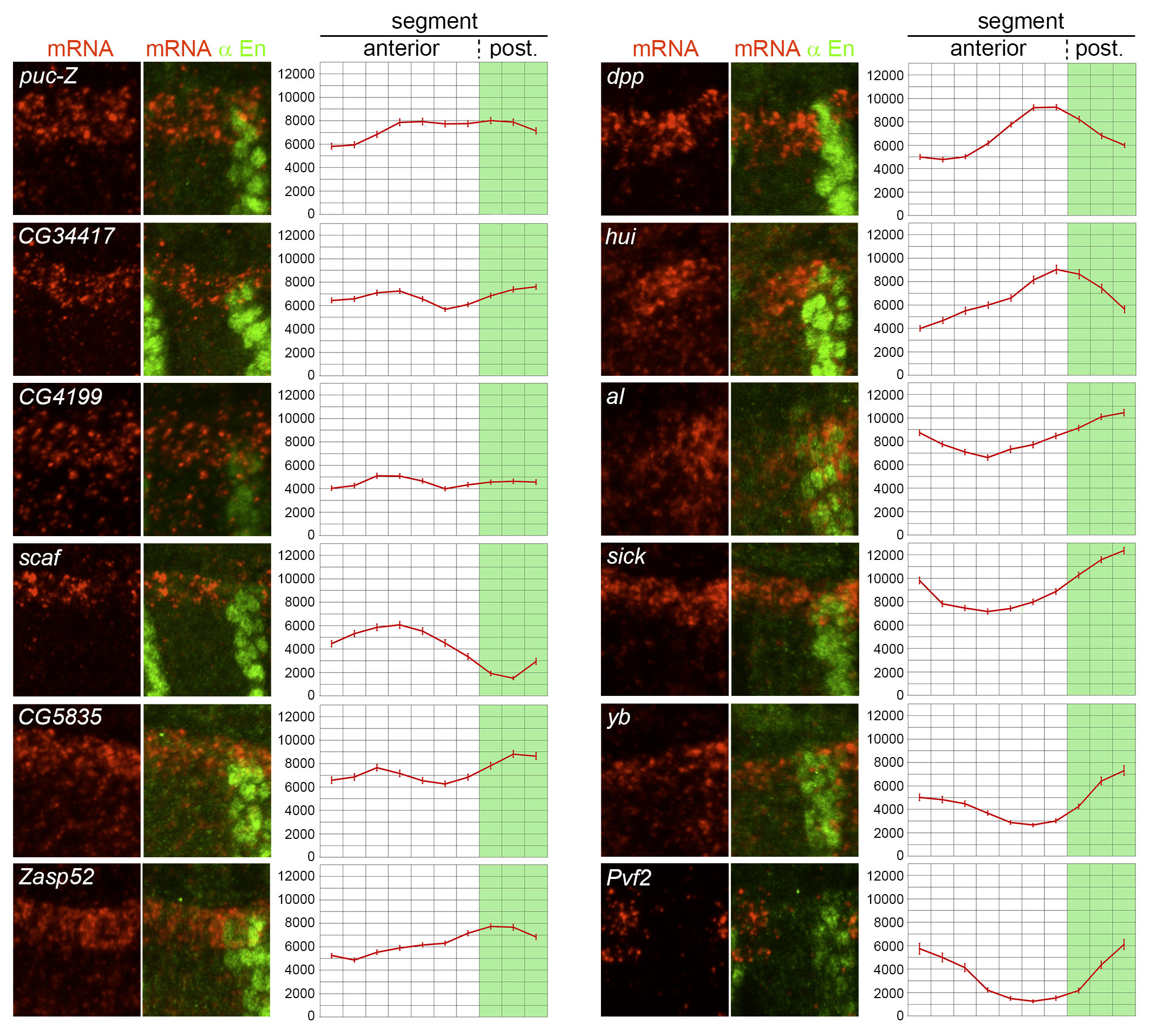

Supplement: S3 Fig — FISH-IF (columns 1, 2, 4 and 5) and mRNA signal quantifications at the LE (columns 3 and 6) are shown for each LE gene. mRNA staining is shown in red alone (columns 1 and 4) or merged with anti-En immuno-staining (in green)(columns 2 and 5). Bar diagram: average mRNA signal intensities at the LE in the ten cells of the segments, expressed as fluorescent intensity (a.u. +/- s.e.m.). This figure is a supplement of Fig 3C. (TIF) [file pgen.1006640.s003.tif]

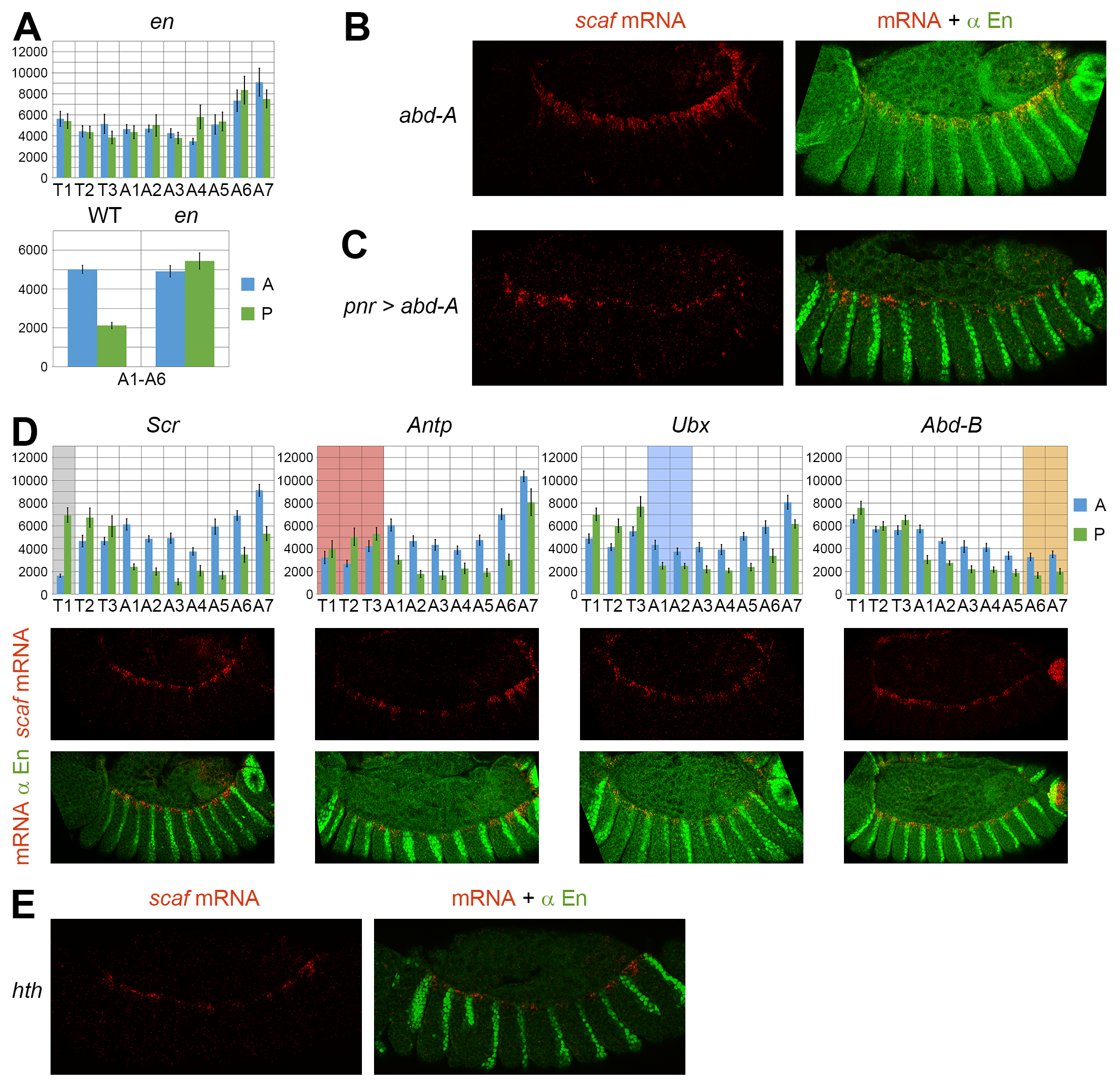

Supplement: S4 Fig — A) Quantification of scaf expression (expressed as fluorescent intensity; a.u. +/- s.e.m.) in the LE of en mutant embryos (n = 10, FISH-IF shown in Fig 4A). The anterior limit for quantification corresponds to the dorsal ridge abutting the head segments, whereas the posterior limit was set just anteriorly to the place where scaf expression starts to expand in the more lateral epidermis (see Fig 4A). The LE was then divided in 10 equal segments for quantification with CurvedPeriodicity. Top panel: quantification in the A (blue) and P (green) compartments along the AP axis. Bottom panel: quantification in the A and P compartments of segments A1 to A6 of the en mutant, compared to WT embryos, showing the absence of negative regulation on scaf expression in the P compartments. B) FISH-IF showing scaf expression (scaf mRNA is in red either merged or not with anti-En staining in green) in the abd-A mutant (quantification shown in Fig 5A). C) FISH-IF in embryos overexpressing abd-A with pnr-GAL4 (quantification shown in Fig 5B). D) scaf expression in Scr (first column), Antp (second column), Ubx (third column) and Abd-B (fourth column) mutants. First row: quantification of scaf expression (expressed as fluorescent intensity; a.u. +/- s.e.m., n = 15 for each mutant) in each compartment (anterior: blue; posterior: green) of each segment along the AP axis. Each main expression domain of the corresponding HOX gene is indicated by a color: grey for Scr, pink for Antp, blue for Ubx and orange for Abd-B. Second row: FISH-IF showing scaf mRNA (red). Third row: FISH-IF showing scaf mRNA (red) with anti-En staining (green). This figure is a supplement of Fig 5D. E) FISH-IF showing scaf expression in the hthP2 mutant (scaf mRNA in red, anti-En staining in green) (quantification shown in Fig 5G). (TIF) [file pgen.1006640.s004.tif]

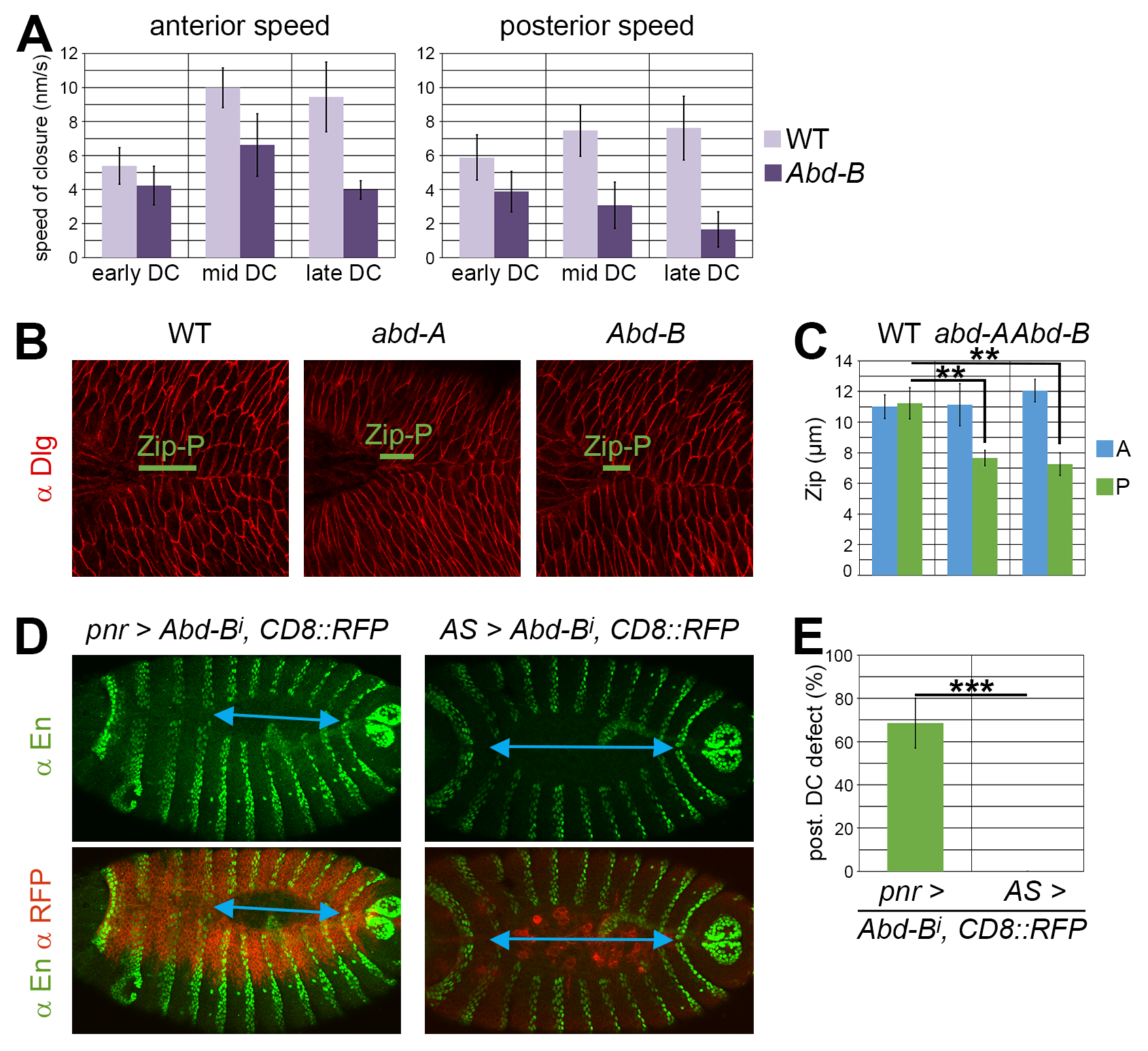

Supplement: S5 Fig — A) Speed of closure (nm/s +/- s.e.m.) in the anterior and posterior regions of control (pink; n = 9) and Abd-B mutant (purple; n = 6) embryos during early DC (seam < 33%), mid DC (33% < seam > 66%) and late DC (seam > 66%). A strong reduction of the posterior closure speed was observed from mid DC (59%) to late DC (78%) in Abd-B embryos. The anterior speed also decreased, especially during late DC (58%). This panel is a supplement of Fig 7D and 7E. B) and C) Dlg-defined zipping zones of WT, abd-A and Abd-B embryos. B) Anti-Dlg (red) immunostainings showing the posterior zipping zones (Zip-P) in the A6 segment of a WT embryo (left) and of abd-A (middle) and Abd-B (right) mutants. The zipping zone is defined by the area where the two opposing LE are in close contact till the formation of a stable septate junction (marked with Dlg). C) Quantification of the anterior (A, blue) and posterior (P, green) zipping zones of the WT embryo and the two HOX mutants. Whereas no variation is observed in the anterior part (no statistical significance), the Zip-P of abd-A (7.7 μm +/- 0.5) and Abd-B (7.3 μm +/- 0.7) are reduced compared to WT embryos (11.2 μm +/- 1.0). As there is no homogeneity of variance for Zip-A, the Kruskal & Wallis test was performed with R, whereas multiple comparisons of Zip-P between WT, abd-A and Abd-B were performed using the Dunnett test (*: p < 0.05; **: p < 0.01; ***: p < 0.001). For WT embryos, n(Zip-A) = 7 and n(Zip-P) = 13; for abd-A, n(Zip-A) = 9 and n(Zip-P) = 14; for Abd-B, n(Zip-A) = 11 and n(Zip-P) = 15. This figure is a supplement of Fig 7F and 7G. D) and E) Inhibition of Abd-B expression in the dorsal ectodermal cells induces posterior DC phenotype. D) Anti-En (green) and anti-RFP (red) immunostainings of embryos expressing Abd-B RNAi (Abd-Bi) and CD8::RFP either with pnr-GAL4 (left panels) or with AS-GAL4 (right panels). Whereas expression in the AS alone has no effect (when A8 closes, T1 is just about to close), expression in the dorsal ecto [file pgen.1006640.s005.tif]
