# Supplementary material for: Signalling crosstalk at the leading edge controls tissue closure dynamics in the Drosophila embryo
Source: PLoS Genet. 2017 Feb 23;13(2):e1006640. doi: 10.1371/journal.pgen.1006640 (PMC5344535; doi:10.1371/journal.pgen.1006640)
Supplement: S1 Table — (DOCX) [file pgen.1006640.s007.docx]

**S1 Table: common genes of the GOF and LOF microarray screen with their corresponding fold change (FC).**

| **Flybase ID** | **Gene** | **Function** | **FC GOF** | **FC LOF** |
| --- | --- | --- | --- | --- |
| FBgn0011706 | reaper | apoptosis | 3,0 | -1,7 |
| FBgn0032725 | Nedd8 | protein degradation tagging | 2,4 | -1,5 |
| FBgn0039923 | Mediator complex sub 26 | transcription factor activity | 1,9 | -1,9 |
| FBgn0043455 | CG5986 | ? | 1,6 | -1,5 |
| FBgn0011828 | Peroxidasin | peroxidase | -1,5 | 1,8 |
| FBgn0004606 | zfh1 | transcription factor | -1,7 | 1,7 |
| FBgn0004396 | CrebA | transcription factor | -2,0 | 1,6 |
