# Supplementary material for: Signalling crosstalk at the leading edge controls tissue closure dynamics in the Drosophila embryo
Source: PLoS Genet. 2017 Feb 23;13(2):e1006640. doi: 10.1371/journal.pgen.1006640 (PMC5344535; doi:10.1371/journal.pgen.1006640)
Supplement: S2 Table — (DOCX) [file pgen.1006640.s008.docx]

**S2 Table: over-represented Gene Ontology (GO) terms in the list of JNK up-regulated genes in the GOF screen (using DAVID).**

| **GO terms** | **N** | **Enrichment Score** |
| --- | --- | --- |
| Cell cycle; cytoskeleton | 11 | 5.5 |
| meiosis | 7 | 5.3 |
| proteolysis | 39 | 4.6 |
| mRNA transport | 5 | 4.5 |
| signalosome | 8 | 4.4 |
| mitosis | 38 | 3.5 |
| mRNA processing | 7 | 3.2 |
| glutathione transferase | 13 | 2.8 |
| ribonucleoprotein complex | 7 | 2.6 |
| nuclear pore complex | 20 | 2.5 |
| eggshell formation | 8 | 2.5 |
| transcription | 16 | 2.5 |
| negative regulation of transcription | 21 | 2.3 |
| nucleotide binding | 7 | 2.3 |
| sexual reproduction | 4 | 2.3 |
| cytoskeleton | 4 | 2.2 |
| oocyte axis specification | 16 | 2.1 |
| protein transport | 7 | 2.0 |
