# Supplementary material for: Signalling crosstalk at the leading edge controls tissue closure dynamics in the Drosophila embryo
Source: PLoS Genet. 2017 Feb 23;13(2):e1006640. doi: 10.1371/journal.pgen.1006640 (PMC5344535; doi:10.1371/journal.pgen.1006640)
Supplement: S3 Table — (DOCX) [file pgen.1006640.s009.docx]

**S3 Table: over-represented Gene Ontology (GO) terms in the list of JNK down-regulated genes of the GOF screen (using DAVID).**

| **GO terms** | **N** | **Enrichment Score** |
| --- | --- | --- |
| regulation of transcription | 5 | 10.3 |
| wing disc morphogenesis | 9 | 9.7 |
| regulation of transcription | 67 | 9.3 |
| wing disc morphogenesis | 6 | 6.7 |
| neuron development | 19 | 6.4 |
| negative regulation of transcription | 6 | 4.2 |
| zinc ion binding | 38 | 4.1 |
| protein kinase | 4 | 2.6 |
| EF hand | 4 | 2.1 |
| endoplasmic reticulum membrane | 4 | 2.0 |
