# Supplementary material for: Signalling crosstalk at the leading edge controls tissue closure dynamics in the Drosophila embryo
Source: PLoS Genet. 2017 Feb 23;13(2):e1006640. doi: 10.1371/journal.pgen.1006640 (PMC5344535; doi:10.1371/journal.pgen.1006640)
Supplement: S4 Table — (DOCX) [file pgen.1006640.s010.docx]

**S4 Table: description of the 31 JNK target genes.**

| **Flybase ID** | **CG** | **Symbol** | **SMART domains (http://smart.embl-heidelberg.de/)** | **FC** | **classification** |
| --- | --- | --- | --- | --- | --- |
| FBgn0038682 | CG5835 | *CG5835* | SP; 1 TM | 2.2 | secreted/membrane |
| FBgn0035770 | CG8588 | *pst* | 3 TM | 2.0 |  |
| FBgn0033968 | CG10200 | *hui* | SP | 2.0 |  |
| FBgn0032601 | CG17914 | *yellow-b* | SP; MRJP (major royal jelly proteins) domain | 2.0 |  |
| FBgn0033033 | CG11066 | *scaf* | SP; Trypsin-like serine protease | 1.8 |  |
| FBgn0035346 | CG1146 | *CG1146* | 1 TM | 1.6 |  |
| FBgn0032135 | CG3881 | *GlcAT-S* | 1 TM; Glycosyltransferase family 43 domain | (1.6) |  |
| FBgn0001291 | CG2275 | *Jra* | BRLZ (bZIP) domain | 1.4 | transcription factor |
| FBgn0000061 | CG3935 | *al* | homeodomain | 1.3 |  |
| FBgn0267698 | CG10295 | *Pak* | P21-Rho-binding domain; Serine/Threonine protein kinase | 1.7 | signalling |
| FBgn0028546 | CG9031 | *ics* | 6 Leucine-rich repeats | 1.5 |  |
| FBgn0044823 | CG14672 | *Spec2* | CRIB (Cdc42-binding) domain | 2.1 |  |
| FBgn0034312 | CG10916 | *CG10916* | Ring finger | 1.6 |  |
| FBgn0031888 | CG13780 | *Pvf2* | SP; PDGF (Platelet-derived and vascular endothelial growth factors) domain | -1.3 |  |
| FBgn0011706 | CG4319 | *rpr* | - (apoptosis) | 3.0 |  |
| FBgn0003328 | CG8095 | *scb* | SP; 7 Integrin alpha domains; 1 TM | (1.5) | cell adhesion |
| FBgn0265991 | CG30084 | *Zasp52* | 2 PDZ domains; LIM domain | (1.1) |  |
| FBgn0031882 | CG9100 | *Rab30* | Rab subfamily of small GTPases | 1.5 | transport |
| FBgn0086346 | CG12876 | *ALiX* | BRO1-like (endosomal targeting) domain | 1.7 |  |
| FBgn0035715 | CG10103 | *CG10103* | IST1 (endosome transport) | 1.5 |  |
| FBgn0030309 | CG1572 | *CG1572* | MARVEL (vesicle trafficking and membrane link) domain; 4 TM | 1.7 |  |
| FBgn0036309 | CG10971 | *Hip1* | ENTH domain (endocytosis); coiled-coil; I/LWEQ domain (actin) | 1.8 | cytoskeleton |
| FBgn0266084 | CG42610 | *Fhos* | 2 Formin Homology 2 domain (actin); Diaphanous GTPase-binding domain | 1.8 |  |
| FBgn0263873 | CG43720 | *sick* | Calponin Homology domain (actin); ATPase | (0.9) |  |
| FBgn0085446 | CG34417 | *CG34417* | Calponin Homology domain (actin) | 1.5 |  |
| FBgn0039869 | CG1890 | *CG1890* | Tubulin chaperone cofactor A | 1.7 |  |
| FBgn0025866 | CG8107 | *CalpB* | 2 EF-hand domains; calpain-type cysteine protease | 1.7 | protease |
| FBgn0027843 | CG6906 | *CAH2* | SP; Eukaryotic-type carbonic anhydrase | 1.7 | enzyme |
| FBgn0063497 | CG17524 | *GstE3* | Glutathione S-transferase | 1.9 |  |
| FBgn0025628 | CG4199 | *CG4199* | Rieske [2Fe-2S] domain; Pyridine nucleotide-disulphide oxidoreductase | 1.7 |  |
| FBgn0037350 | CG2911 | *CG2911* | DnaJ chaperone homology domain; CSL zinc finger (intron *Spec2*) | 1.6 | chaperone |
